# Supplementary material for: Exposure to circadian disrupting environment and high-fat diet during pregnancy and lactation alter reproductive competence and lipid profiles of liver, mammary, plasma and milk of ICR mice
Source: PLoS One. 2025 Mar 31;20(3):e0320538. doi: 10.1371/journal.pone.0320538 (PMC11957368; doi:10.1371/journal.pone.0320538)
Supplement: S2 File — (DOCX) [file pone.0320538.s002.docx]

**Exposure to circadian disrupting environment and high-fat diet during pregnancy and lactation alter reproductive competence and lipid profiles of liver, mammary, plasma and milk of ICR mice**

Leriana Garcia Reis^1^, Kelsey Teeple^1^, Michayla Dinn^1^, Jenna Schoonmaker^1^, Sara Scinto^1^, Christina Ramires Ferreira^2^, and Theresa Casey^1*^

Running head: Effects of circadian disruption and obesity on maternal and offspring health

^1^Purdue University, Department of Animal Science, West Lafayette, Indiana 47907, USA.

^2^Bindley Bioscience Center, Purdue University, West Lafayette, IN, USA.

*Corresponding author: Theresa Casey. Phone number: +1 (800) 373-1319

Email: theresa-casey@purdue.edu

Key words: circadian rhythm, phase-shift light exposure, lactation, gestation, chronic light-dark phase circadian disruption, maternal physiology

Table 1. Effects of diet, light, physiological stage, and period of day on mice feed intake and energy intake during pregnancy and lactation

|  | **Diet (± SEM)** | | **Light (± SEM)** | | **Stage (± SEM)** | | | | | | **Period of Day (± SEM)** | |
| --- | --- | --- | --- | --- | --- | --- | --- | --- | --- | --- | --- | --- |
|  | **CON** | **HF** | **LD** | **PS** | **Gestation d1-6** | **Gestation d7-13** | **Gestation d14-18** | **Lactation d1-4** | **Lactation d5-8** | **Lactation d9-12** | **Day** | **Night** |
| Feed intake, g | 4.23 ± 0.10 | 3.20 ± 0.11 | 3.57 ± 0.09 | 3.86 ± 0.12 | 1.74 ± 0.21 | 2.00 ± 0.14 | 2.62 ± 0.13 | 3.84 ± 0.13 | 5.58 ± 0.14 | 6.53 ± 0.14 | 2.28 ± 0.096 | 5.16 ± 0.095 |
| Feed intake, kcal | 16.32 ± 0.47 | 16.82 ± 0.51 | 16 ± 0.41 | 17.14 ± 0.56 | 7.88 ± 0.9 | 8.93 ± 0.59 | 11.64 ± 0.58 | 17.06 ± 0.58 | 24.8 ± 0.58 | 29.12 ± 0.6 | 10.21 ± 0.03 | 22.94 ± 0.03 |

|  | ***P-value*** | | | | | | | | | | |
| --- | --- | --- | --- | --- | --- | --- | --- | --- | --- | --- | --- |
|  | **Diet** | **Light** | **Period of Day** | **Stage** | **Diet*Light** | **Diet*Stage** | **Diet*Period of Day** | **Light*Stage** | **Light*Period of Day** | **Stage*Period of Day** | **Stage*Light*Period of Day** |
| Feed intake, g | **<0.01** | **<0.01** | **<0.01** | **<0.01** | 0.09 | 0.59 | 0.10 | 0.94 | **<0.01** | 0.09 | **0.05** |
| Feed intake, kcal | 0.20 | **<0.01** | **<0.01** | **<0.01** | 0.22 | 0.49 | 0.43 | 0.67 | **<0.01** | 0.18 | **<0.04** |

Table 2. Effects of diet, light, physiological stage, and period of day on mice corticosterone level and fecal weight during pregnancy and lactation

|  | Diet (± SEM) | | Light (± SEM) | | Stage (± SEM) | | | Period of Day (± SEM) | |
| --- | --- | --- | --- | --- | --- | --- | --- | --- | --- |
|  | CON | HF | LD | PS | Early prgnancy | Late pregnancy | Lactation | Day | Night |
| Corticosterone, pg.mL^-1^ | 5630.87 ± 8048.44 | 5541.88 ± 8031.11 | 5568.30 ± 8045.36 | 5604.45 ± 8034.19 | 5512.38 ± 8079.15 | 6185.12 ± 8031.86 | 5061.63 ± 8010.29 | 5543.57 ± 8047.00 | 5629.18 ± 8032.56 |
| Feces weight, g | 0.310 ± 0.034 | 0.380 ± 0.029 | 0.270 ± 0.031 | 0.420 ± 0.031 | 0.400 ± 0.035 | 0.340 ± 0.043 | 0.290 ± 0.036 | 0.340 ± 0.031 | 0.350 ± 0.031 |
| Corticosterone output, pg.g^-1^ | 3023.07 ± 479.51 | 2688.35 ± 428.15 | 2285.26 ± 460.46 | 3426.16 ± 445.60 | 2907.41 ± 583.87 | 3643.42 ± 662.59 | 2016.31 ± 673.29 | 2379.65 ± 553.57 | 3331.77 ± 499.03 |

|  | ***P-value*** | | | | | | | | | | | | | |
| --- | --- | --- | --- | --- | --- | --- | --- | --- | --- | --- | --- | --- | --- | --- |
|  | **Diet** | **Light** | **Stage** | **Day** | **Diet*Light** | **Diet*Stage** | **Light*Stage** | **Diet*Day** | **Light*Day** | **Stage*Day** | **Diet*Light*Stage** | **Diet*Light*Day** | **Diet*Stage*Day** | **Light*Stage*Day** |
| Corticosterone, pg.mL^-1^ | 0.681 | 0.540 | **0.0003** | 0.643 | 0.736 | 0.720 | **0.004** | 0.709 | 0.213 | 0.903 | 0.590 | 0.862 | 0.976 | 0.913 |
| Feces weight, g | 0.155 | **0.001** | 0.106 | 0.859 | **0.008** | 0.364 | 0.543 | 0.933 | 0.961 | 0.341 | 0.605 | 0.746 | 0.579 | 0.420 |
| Corticosterone output, pg.g^-1^ | 0.876 | **0.017** | **0.007** | 0.495 | 0.114 | 0.319 | 0.240 | 0.671 | **0.068** | 0.926 | 0.975 | 0.722 | 0.789 | 0.460 |

Table 3. Effect of diet and light on dam’s weight, prolactin, TAG, and HbA1c levels

|  | **Diet** | | **Light** | | ***P-value*** | | |
| --- | --- | --- | --- | --- | --- | --- | --- |
|  | **CON** | **HF** | **LD** | **PS** | **Diet** | **Light** | **Diet*Light** |
| Serum Prolactin, pg.m^-1^ | 122.79 ± 11.58 | 136.98 ± 11.16 | 138.2 ± 11.58 | 121.56 ± 11.16 | 0.387 | 0.312 | 0.830 |
| Serum TAG, mg.dL^-1^ | 85.40 ± 6.96 | 83.30 ± 7.74 | 90.67 ± 7.51 | 78.03 ± 7.20 | 0.842 | 0.236 | 0.685 |
| HbA1c, % | 2.50 ± 0.11 | 2.71 ± 0.12 | 2.67 ± 0.12 | 2.54 ± 0.12 | 0.210 | 0.463 | 0.432 |
| Dam weight, g | 30.62 ± 0.72 | 32.79 ± 0.73 | 31.89 ± 0.72 | 31.52 ± 0.72 | **0.041** | 0.717 | 0.371 |

Table 4. Impact of diet and light on milk composition of dams: TAG, protein, and lactose levels

|  | **Diet** | | **Light** | | ***P-value*** | | |
| --- | --- | --- | --- | --- | --- | --- | --- |
|  | **CON** | **HF** | **LD** | **PS** | **Diet** | **Light** | **Diet*Light** |
| Lactose, ng.mL^-1^ | 3801.37 ± 173.43 | 4609.94 ± 210.03 | 4353.32 ± 179.12 | 4057.99 ± 205.20 | **0.007** | 0.290 | **0.037** |
| Protein, mg.mL^-1^ | 71.87 ± 3.13 | 75.19 ± 3.54 | 71.97 ± 3.24 | 75.09 ± 3.45 | 0.490 | 0.520 | 0.851 |
| TAG, mg.dL^-1^ | 4076.27 ± 384.28 | 3336.71 ± 434.76 | 3769.4 ± 396.88 | 3643.58 ± 423.29 | 0.215 | 0.830 | 0.472 |

Table 5. Effect of maternal diet and PS exposure on birth litter size, weigh suckle weigh, and litter weight

|  | Diet | | Light | | *P-value* | | |
| --- | --- | --- | --- | --- | --- | --- | --- |
|  | CON | HF | LD | PS | Diet | Light | Diet*Light |
| Birth litter size, n | 11.56 ± 0.44 | 11.92 ± 0.48 | 11.69 ± 0.44 | 11.79 ± 0.48 | 0.593 | 0.875 | 0.682 |
| Weigh suckle weigh, g | 1.38 ± 0.20 | 1.73 ± 0.21 | 1.55 ± 0.19 | 1.55 ± 0.21 | 0.223 | 0.990 | 0.130 |
| Litter weight, g | 40.97 ± 0.86 | 47.09 ± 0.68 | 43.41 ± 0.57 | 44.65 ± 0.94 | **<0.001** | 0.260 | 0.230 |
