# Supplementary material for: Exposure to circadian disrupting environment and high-fat diet during pregnancy and lactation alter reproductive competence and lipid profiles of liver, mammary, plasma and milk of ICR mice
Source: PLoS One. 2025 Mar 31;20(3):e0320538. doi: 10.1371/journal.pone.0320538 (PMC11957368; doi:10.1371/journal.pone.0320538)
Supplement: S4 File — (DOCX) [file pone.0320538.s004.docx]

**The impact of circadian disruption and high-fat diet on ICR mice reproductive competence, fecal corticosterone and lipid profiles in mid-lactation: implications for maternal-neonate health**

Leriana Garcia Reis^1^, Kelsey Teeple^1^, Michayla Dinn^1^, Jenna Schoonmaker^1^, Sara Scinto^1^, Christina Ramires Ferreira^2^, and Theresa Casey^1*^

Running head: Effects of circadian disruption and obesity on maternal and offspring health

^1^Purdue University, Department of Animal Science, West Lafayette, Indiana 47907, USA.

^2^Bindley Bioscience Center, Purdue University, West Lafayette, IN, USA.

*Corresponding author: Theresa Casey. Phone number: +1 (800) 373-1319

Email: theresa-casey@purdue.edu

Key words: circadian rhythm, phase-shift light exposure, lactation, gestation, chronic light-dark phase circadian disruption, maternal physiology

PS treatment downregulated the relative abundance of 25.4% of the 67 lipids modified in plasma of CON mice, included PE, PC, PS, and TG (Fig. 1). Conversely, 74.6% of the lipids were upregulated, consisting of PE, PC, PS, PG, TG, and SM.

**Figure 1. Distribution of downregulated and upregulated lipids in mice plasma under PS light treatment within the CON diet.**

The liver, being the third most affected matrix by PS light exposure within CON diet, showed that 31.1% of the lipid classes (TG, PC, PE, PG, PS, and SM) were downregulated (Fig. 2), while 62.9% of the lipids (TG, CE, DG, FA, PC, PE, PG, and PI) were upregulated (Fig. 2).

**Figure 2. Distribution of downregulated and upregulated lipids in mice liver under PS light treatment within the CON diet**

In turn, of the 6 significant lipids modified in milk by exposure to PS light in CON diet, 50% were downregulated, including DG, PS, and PG (Fig. 3). Conversely, PS light upregulated the other 50% of lipids, which consisted of the TG and PC classes (Fig. 3).

**Figure 3. Distribution of downregulated and upregulated lipids in mice milk under PS light treatment within the CON diet.**
