# Supplementary material for: Exposure to circadian disrupting environment and high-fat diet during pregnancy and lactation alter reproductive competence and lipid profiles of liver, mammary, plasma and milk of ICR mice
Source: PLoS One. 2025 Mar 31;20(3):e0320538. doi: 10.1371/journal.pone.0320538 (PMC11957368; doi:10.1371/journal.pone.0320538)
Supplement: Attachment — Submitted filename: PONE-D-24-38949_reviewer-2.pdf [file pone.0320538.s005.pdf]

PLOS ONE

The impact of circadian disruption and high-fat diet on ICR mice reproductive competence, fecal corticosterone and lipid profiles in mid-lactation: implications for maternal-neonate health

--Manuscript Draft--

**Manuscript Number:**	PONE-D-24-38949
**Article Type:**	Research Article
**Full Title:**	The impact of circadian disruption and high-fat diet on ICR mice reproductive competence, fecal corticosterone and lipid profiles in mid-lactation: implications for maternal-neonate health
**Short Title:**	Effects of circadian disruption and obesity on maternal and offspring health
**Corresponding Author:**	Theresa Casey, PhD<br>Purdue University<br>West Lafayette, Indiana UNITED STATES OF AMERICA
**Keywords:**	circadian rhythm; phase-shift light exposure; lactation; gestation; chronic light-dark phase circadian disruption; maternal physiology
**Abstract:**	Obesity and exposure to circadian-disrupting environments have been linked to reproductive dysfunction, subfertility, and poor breastfeeding outcomes in women. We investigated the effects of pre-pregnancy obesity induced by a high-fat diet and exposure to circadian-disrupting light-dark phase shifts on birth littler size, pup survival to 24h, growth to lactation day 12, and their relationship to maternal feeding patterns, fecal corticosterone levels, milk composition, and lipid profiles of liver, plasma, mammary gland, and milk. Female ICR mice, three-weeks-old, were assigned to control (CON; 10% fat) or high fat (HF; 60% fat) diet for four weeks to establish body mass differentiation before breeding. After mating, the mice were assigned a 12-hour light-dark (LD) cycle or a chronic 6-hour phase-shifted (PS) light-dark cycle until lactation day 12 (L12). Diurnal feed intake and fecal corticosterone were monitored throughout the study. On L12, circulating levels of prolactin, glycosylated hemoglobin (HbA1c), and triacylglycerols (TG) were measured in maternal blood. Milk yield was assessed with weigh-suckle-weigh on L10, and lactose, TG, and protein levels in milk were measured on L12. Exposure to PS increased maternal feed intake and fecal corticosterone levels across physiological states (P<0.05). The HF diet increased pre-pregnancy body weight and elevated lactose levels in milk compared to the CON diet group, while PS treatment reduced milk lactose levels in the CON diet group. Additionally, the HF diet increased the carbon length of diacylglycerols (DG) in the liver and milk, and of TG in plasma, mammary gland and milk. HF also increased the carbon chain length of phosphatidylcholine (PC) in plasma, with a general increase in the number of double bonds across these lipid classes. These findings suggest a profound metabolic influence of diet and light exposure on feeding behavior and stress responses, indicating potential circadian influences on parturition timing. Alterations in lipid profiles indicate HF and PS exposure may increase oxidative damage to lipids and thus potentially impact their functions having implications in maternal health and neonate development.
**Order of Authors:**	Leriana Garcia Reis, Ph.D.
	Kelsey Teeple
	Michayla Dinn
	Jenna Schoonmaker
	Sara Brooke Scinto
	Christina Ramires Ferreira
	Theresa Casey
**Additional Information:**	
**Question**	**Response**

Powered by Editorial Manager® and ProduXion Manager® from Aries Systems Corporation

**Financial Disclosure**<br><br>Enter a financial disclosure statement that describes the sources of funding for the work included in this submission. Review the submission guidelines for detailed requirements. View published research articles from *PLOS ONE* for specific examples.<br><br>This statement is required for submission and **will appear in the published article** if the submission is accepted. Please make sure it is accurate.<br><br>**Funded studies**<br>Enter a statement with the following details:<br>• Initials of the authors who received each award<br>• Grant numbers awarded to each author<br>• The full name of each funder<br>• URL of each funder website<br>• Did the sponsors or funders play any role in the study design, data collection and analysis, decision to publish, or preparation of the manuscript?<br><br>Did you receive funding for this work?	Yes
Please add funding details.<br>as follow-up to "**Financial Disclosure**<br><br>Enter a financial disclosure statement that describes the sources of funding for the work included in this submission. Review the submission guidelines for detailed requirements. View published research articles from *PLOS ONE* for specific examples.<br><br>This statement is required for submission and **will appear in the published article** if the submission is accepted. Please make sure it is accurate.	The authors would like to thank the São Paulo Research Foundation (FAPESP) by the financial support given to Leriana Garcia Reis (Grant nº 2021/12819-0). The funders had no role in study design, data collection and analysis, decision to publish, or preparation of the manuscript.<br>https://fapesp.br/en

**Funded studies**<br>Enter a statement with the following details:<br>• Initials of the authors who received each award<br>• Grant numbers awarded to each author<br>• The full name of each funder<br>• URL of each funder website<br>• Did the sponsors or funders play any role in the study design, data collection and analysis, decision to publish, or preparation of the manuscript?<br><br>Did you receive funding for this work?"	
Please select the country of your main research funder (please select carefully as in some cases this is used in fee calculation).<br>as follow-up to "**Financial Disclosure**<br><br>Enter a financial disclosure statement that describes the sources of funding for the work included in this submission. Review the submission guidelines for detailed requirements. View published research articles from *PLOS ONE* for specific examples.<br><br>This statement is required for submission and **will appear in the published article** if the submission is accepted. Please make sure it is accurate.<br><br>**Funded studies**<br>Enter a statement with the following details:<br>• Initials of the authors who received each award<br>• Grant numbers awarded to each author<br>• The full name of each funder<br>• URL of each funder website<br>• Did the sponsors or funders play any role in the study design, data collection and analysis, decision to publish, or preparation of the manuscript?<br><br>Did you receive funding for this work?"	BRAZIL - BR
**Competing Interests**	The authors have declared that no competing interests exist

Use the instructions below to enter a competing interest statement for this submission. On behalf of all authors, disclose any competing interests that could be perceived to bias this work—acknowledging all financial support and any other relevant financial or non-financial competing interests.<br><br>This statement is required for submission and **will appear in the published article** if the submission is accepted. Please make sure it is accurate and that any funding sources listed in your Funding Information later in the submission form are also declared in your Financial Disclosure statement.<br><br>View published research articles from *PLOS ONE* for specific examples.<br><br>**NO authors have competing interests**<br><br>Enter: *The authors have declared that no competing interests exist.*<br><br>**Authors with competing interests**<br><br>Enter competing interest details beginning with this statement:<br><br>*I have read the journal's policy and the authors of this manuscript have the following competing interests: [insert competing interests here]*<br><br>* typeset	
**Ethics Statement**<br><br>Enter an ethics statement for this submission. This statement is required if the study involved:<br><br>• Human participants<br>• Human specimens or tissue<br>• Vertebrate animals or cephalopods<br>• Vertebrate embryos or tissues	Animal use protocols were reviewed and approved by the Institutional Animal Care and Use Committee (IACUC; protocol # 2104002135) of Purdue University prior to beginning studies.

• Field research<br><br>Write "N/A" if the submission does not require an ethics statement.<br><br>General guidance is provided below. Consult the submission guidelines for detailed instructions. **Make sure that all information entered here is included in the Methods section of the manuscript.**	

Powered by Editorial Manager® and ProduXion Manager® from Aries Systems Corporation

**Format for specific study types**<br><br>**Human Subject Research (involving human participants and/or tissue)**<br>• Give the name of the institutional review board or ethics committee that approved the study<br>• Include the approval number and/or a statement indicating approval of this research<br>• Indicate the form of consent obtained (written/oral) or the reason that consent was not obtained (e.g. the data were analyzed anonymously)<br><br>**Animal Research (involving vertebrate animals, embryos or tissues)**<br>• Provide the name of the Institutional Animal Care and Use Committee (IACUC) or other relevant ethics board that reviewed the study protocol, and indicate whether they approved this research or granted a formal waiver of ethical approval<br>• Include an approval number if one was obtained<br>• If the study involved *non-human primates*, add *additional details* about animal welfare and steps taken to ameliorate suffering<br>• If anesthesia, euthanasia, or any kind of animal sacrifice is part of the study, include briefly which substances and/or methods were applied<br><br>**Field Research**<br><br>Include the following details if this study involves the collection of plant, animal, or other materials from a natural setting:<br>• Field permit number<br>• Name of the institution or relevant body that granted permission	
**Data Availability**<br><br>Authors are required to make all data underlying the findings described fully available, without restriction, and from the time of publication. PLOS allows rare exceptions to address legal and ethical concerns. See the PLOS Data Policy and FAQ for detailed information.	Yes - all data are fully available without restriction

Powered by Editorial Manager® and ProduXion Manager® from Aries Systems Corporation

A Data Availability Statement describing where the data can be found is required at submission. Your answers to this question constitute the Data Availability Statement and **will be published in the article**, if accepted.<br><br>**Important:** Stating 'data available on request from the author' is not sufficient. If your data are only available upon request, select 'No' for the first question and explain your exceptional situation in the text box.<br><br>Do the authors confirm that all data underlying the findings described in their manuscript are fully available without restriction?	
**Describe where the data may be found in full sentences. If you are copying our sample text, replace any instances of XXX with the appropriate details.**<br><br>• If the data are **held or will be held in a public repository**, include URLs, accession numbers or DOIs. If this information will only be available after acceptance, indicate this by ticking the box below. For example: *All XXX files are available from the XXX database (accession number(s) XXX, XXX.).*<br>• If the data are all contained **within the manuscript and/or Supporting Information files**, enter the following: *All relevant data are within the manuscript and its Supporting Information files.*<br>• If neither of these applies but you are able to provide **details of access elsewhere**, with or without limitations, please do so. For example:<br><br>*Data cannot be shared publicly because of [XXX]. Data are available from the XXX Institutional Data Access / Ethics Committee (contact via XXX) for researchers who meet the criteria for access to confidential data.*<br><br>*The data underlying the results presented in the study are available from (include the name of the third party*	All relevant data are within the manuscript and its Supporting Information files.

Powered by Editorial Manager® and ProduXion Manager® from Aries Systems Corporation

and contact information or URL).<br>• This text is appropriate if the data are owned by a third party and authors do not have permission to share the data.<br><br>* typeset	
Additional data availability information:	

Powered by Editorial Manager® and ProduXion Manager® from Aries Systems Corporation

Manuscript

Click here to access/download;Manuscript;Manuscript_09_04_2024.doc

The impact of circadian disruption and high-fat diet on ICR mice reproductive competence, fecal corticosterone and lipid profiles in mid-lactation: implications for maternal-neonate health

Leriana Garcia Reis[1], Kelsey Teeple[1], Michayla Dinn[1], Jenna Schoonmaker[1], Sara Scinto[1], Christina Ramires Ferreira[2], and Theresa Casey[1*]

Running head: Effects of circadian disruption and obesity on maternal and offspring health

[1]Purdue University, Department of Animal Science, West Lafayette, Indiana 47907, USA.

[2]Bindley Bioscience Center, Purdue University, West Lafayette, IN, USA.

*Corresponding author: Theresa Casey. Phone number: +1 (800) 373-1319

Email: theresa-casey@purdue.edu

Key words: circadian rhythm, phase-shift light exposure, lactation, gestation, chronic light-dark phase circadian disruption, maternal physiology

Abstract

Obesity and exposure to circadian-disrupting environments have been linked to reproductive dysfunction, subfertility, and poor breastfeeding outcomes in women. We investigated the effects of pre-pregnancy obesity induced by a high-fat diet and exposure to circadian-disrupting light-dark phase shifts on birth littler size, pup survival to 24h, growth to lactation day 12, and their relationship to maternal feeding patterns, fecal corticosterone levels, milk composition, and lipid profiles of liver, plasma, mammary gland, and milk. Female ICR mice, three-weeks-old, were assigned to control (CON; 10% fat) or high fat (HF; 60% fat) diet for four weeks to establish body mass differentiation before breeding. After mating, the mice were assigned a 12-hour light-dark (LD) cycle or a chronic 6-hour phase-shifted (PS) light-dark cycle until lactation day 12 (L12). Diurnal feed intake and fecal corticosterone were monitored throughout the study. On L12, circulating levels of prolactin, glycosylated hemoglobin (HbA1c), and triacylglycerols (TG) were measured in maternal blood. Milk yield was assessed with weigh-suckle-weigh on L10, and lactose, TG, and protein levels in milk were measured on L12. Exposure to PS increased maternal feed intake and fecal corticosterone levels across physiological states ($P<0.05$). The HF diet increased pre-pregnancy body weight and elevated lactose levels in milk compared to the CON diet group, while PS treatment reduced milk lactose levels in the CON diet group. Additionally, the HF diet increased the carbon length of diacylglycerols (DG) in the liver and milk, and of TG in plasma, mammary gland and milk. HF also increased the carbon chain length of phosphatidylcholine (PC) in plasma, with a general increase in the number of double bonds across these lipid classes. These findings suggest a profound metabolic influence of diet and light exposure on feeding behavior and stress responses, indicating potential circadian influences on parturition timing. Alterations in lipid profiles indicate HF and PS exposure may increase oxidative damage to lipids and thus potentially impact their functions having implications in maternal health and neonate development.

Introduction

Obesity and circadian disrupting environments such rotating shift work, transmeridian travel, and behaviors that cause social jetlag are common in modern societies [1]. Epidemiological evidence links obesity and circadian disruption to the development of chronic non-communicable diseases like metabolic syndrome and cancer [2,3]. Obesity and exposure to circadian disrupting environments are also linked to reproductive dysfunction, subfertility, and poorer breastfeeding outcomes in women [4–6]. The integrated and reciprocal regulation of the circadian and metabolic systems likely confound the influence of obesity and circadian disruption on the reproductive competence of women. Both are related to development of insulin resistance and dyslipidemia and alter hormonal milieu, all of which are linked to decreased fertility and poorer lactation outcomes in humans [5].

Circadian rhythms are 24 h cycles of behavior and physiology that function to synchronize physiological processes across the body and with the external environment. Circadian clocks that generate these rhythms play a central role in maintaining metabolic homeostasis and coordinating the timing of reproductive processes like ovulation and parturition [7–10]. The 24 h rhythms generated by circadian clocks are integrated into homeostatic feedback loops and repair pathways. Responsiveness of the master clock in the suprachiasmatic nuclei (SCN) to light and peripheral clocks to feeding time enables synchronization and adaptation to seasonal changes in day length and food availability. However, exogenous and endogenous factors that function as inputs to the circadian clocks disrupt their timing and the order of physiologic functions when applied at unusual and inappropriate times and are referred to as chronodisruptors [11]. Changes in the natural light-dark cycle perturb the circadian system. Other chronodisrupters include inappropriately timed food intake and physical activity and biological stress [9–11]. Nutritional quality and obesity also play a role in maintaining robustness of the circadian system.

Orchestrated changes occur in maternal behavior and physiology to support the growth and development of the fetus during gestation and the nutritional demands of neonates during lactation. Maternal nutritional state and the external environment can impact these adaptions to pregnancy and lactation [12]. Since maternal environment governs offspring environment during pregnancy and lactation, these alterations can have profound and long-term effects on offspring development and health. For example, exposure of pregnant ewes to a changing shift work model altered maternal metabolism and increased gestation length and decreased birth weight of offspring [13]. Studies of rats found maternal exposure to chronic light-phase shifts during gestation and the first week postnatal resulted in rat offspring displaying age and gender dependent hyperleptinemia, hyperinsulinemia, poor glucose tolerance, insulin resistance and increased adiposity [14]. Exposure of offspring to maternal circadian disruption during lactation alone reduced adult body mass, increased social avoidance and hyperactivity. Whereas exclusive *in utero* exposure to maternal circadian disruption was associated with social

avoidance and hyperactivity [15]. Epidemiologic data and controlled animal studies demonstrate that early exposure to maternal high-fat diet (HFD) during pregnancy and lactation also has long-term effects on offspring health, which include development of metabolic syndrome, hypertension, type 2 diabetes, obesity, cardiovascular dysfunction and alterations in neurological development [16–20].

Our overall goal is to determine if and how disrupting circadian clocks and obesity interact to affect maternal reproductive competence and the consequence to offspring. Our previous studies found that feeding a high fat diet for 4 weeks prior to mating significantly increased body weight and percent body fat of female mice as well as altered their circadian feeding patterns, attenuated corticosterone circadian rhythms and significantly increased hair corticosterone levels, independent of final body weight [21]. Here we describe a 2 by 2 factorial designed study to analyze the effect of circadian disruption through exposure to continuous 6h light-dark phase shifts and high fat diet induced obesity during gestation and lactation on birth litter size and postnatal growth to lactation day 12. These findings were related to the effect of treatment and diet on maternal diurnal and nocturnal feed intake and fecal corticosterone output across pregnancy and lactation, circulating prolactin, triacylglycerol (TG) and Hb1Ac levels, an indicator of glycemic control, and milk macronutrient composition. In addition, our previous study found that high fat diet increased the levels of malondialdehyde (MDA) in milk. MDA is a marker if oxidative damage to lipids via peroxidation. High fat diet and circadian disruption increase oxidative stress and dyslipidemia [22,23], and thus we conducted exploratory lipidome analysis using multiple reaction monitoring (MRM) to determine if treatment and diet affected liver, plasma, mammary gland and milk lipid profiles. We hypothesized that high fat diet and exposure to PS would increase oxidative stress. Therefore, lipid profiles of tissues and matrices (blood and milk) would have a greater proportion of shorter fatty acid chain lengths in HF and PS animals relative to control and LD treated mice. Moreover, our previous studies found that HF increases the chain length of circulating and milk lipids indicative of fatty acids being sourced primarily from diet or adipose stores rather than *de novo* synthesized in the mammary gland. Thus, we predicted a shift in lipids in HF fed animals to have both shorter and longer carbon chain lengths.

Material and methods

Animals and experimental design

Animal use protocols were reviewed and approved by the Institutional Animal Care and Use Committee (IACUC; protocol # 2104002135) of Purdue University prior to beginning studies. Three-week-old female ICR mice (n=40) were obtained from Evigno (CD1, Indianapolis, IN, USA), ear tagged, acclimated for two weeks, and then randomly assigned into one of two dietary treatments: control (CON, n=20) and high fat (HF, n=20).

More females were assigned to HF treatment to account for lower expected fertility of animals on this diet [24]. Prior to mating, the females were housed in groups of 5 based on treatment for 4 weeks and fed either a control diet (Research Diets D12450J, 10% of total kcal energy is fat and 7% of total kcal is sucrose; 3.85 kcal/g) or high fat diet (Research Diets D12492, 60% of kcal energy is fat and 7% of kcal energy is sucrose; 5.24 kcal/g), as we have described [21]. During acclimation and the following four-week period all mice were exposed to 12 h light-12 h dark cycles.

After the 4-weeks on experimental diets, females were weighed (pre-pregnancy weight) and mated with males at a 2 to 1 ratio. Females were checked twice daily at 12 h intervals for the presence of a vaginal plug, indicating successful mating and considered gestation day 1. Upon observation of vaginal plug, females were moved into one of the experimental light rooms where they were singularly housed and remained until 12 days postpartum. The experimental lighting was control (LD), or phase shifted (PS). The LD mice were exposed to regular 12 h light (L) - 12 h dark (D) cycles, with the light phase averaging 114 lux at mouse eye level. The PS mice were exposed to the same lux of light for 12 h each day, and 12 h of darkness, but every three days the start of the light cycle was shifted forward 6 h (Fig 1). Although mice were checked twice daily for vaginal plugs after placement with males for mating, the presence was only observed in 18 of the 40 mice and there were not enough animals in each treatment group to analyze the effect of diet and light on length of gestation. The 2 X 2 factorial study design resulted in 4 treatments of CON-LD (n=8), CON-PS (n=9), HF-LD (n=8) and HF-PS (n=7).

Fig 1. Experimental design and data collection overview of the 2 x 2 factorial study.

Females were allowed to birth naturally, time of day of birth was recorded and at 24 h postnatal, litters were standardized to n=8 neonates/dam. Birth litter size, and pup survival to 24 h were recorded. Litter weight was taken every 2 days from day 0 to day 12 of lactation, dams and pups were separated at 0600. Time is expressed in a 24-hour format (e.g., 0600 indicates 6:00 AM). Pups were weighed. After 3 h of separation from pups, dams were milked as previously described [21] under light anesthesia using 3% isoflurane gas at a rate of 1.0 L/minute oxygen. To facilitate milk let down, dams were administered 0.2 mL of oxytocin (IP 20 Units/mL, Vet One, Boise, ID USA). Immediately after milking, dams were euthanized via slow fill CO_2 inhalation, followed by cervical dislocation as a secondary method. Blood was collected from the dam via cardiac punction and placed in an 7.5% EDTA K3 plasma separating tube (Covidien, Dublin, Ireland). Dams were then weighed.

Weigh-suckle-weigh to estimate milk production and litter parameters

On lactation d10, dam was removed from cage for 2 h between 0800-1000. Time is expressed in a 24-hour format (e.g., 0800 indicates 8:00 AM). The cage was placed on a heating pad to keep pups warm. At the end of 2 h separation, pups were weighed and then dam was returned to the cage and pups were observed

suckling and then weighed. The difference in weight from prior to and then after suckling was used to estimate milk production. Birth litter size, litter weight, time of day of birth and pup survival to 24 h were also evaluated.

Milk composition analysis

Milk was thawed overnight at 4˚C under constant rotation to homogenize. Milk was diluted 1:100 with PBS for analysis of protein content using a Bradford assay (Fisher Scientific, Waltham, MA, USA) and 1:500 with PBS to measure lactose using a commercial kit (MAK017, Sigma-Aldrich, St. Louis, MO, USA). Milk TG was measured by preparing a 1:5 dilution using the Cayman Chemical kit (Ann Arbor, MI, USA).

Blood and plasma analyses

Glycated hemoglobin (HbA1c) is a validated marker of glycemic control in mice [25–27]. Whole blood HbA1c was measured in undiluted whole blood collected in EDTA tubes using a commercial assay kit (Crystal Chem, Elk Grove Village, IL, USA). Plasma triacylglycerol (TG) was measured following 1:5 dilution with PBS using the Cayman Chemical kit. Plasma prolactin was measured in a 1:20 dilution of plasma using an ELISA kit (ab100736, Abcam, Waltham, MA, USA). All assays were performed following the manufacturers' instructions.

Total feed intake

Feed intake was measured for the period of the day (0600–1800) and night (1800–0600). Intake was determined by weighing feed at 0600 and 1800 daily. To calculate the amount of food consumed during the day, the amount (grams) of food the mice had at 1800 was subtracted from the amount of food they started with at 0600 (0600 - 1800). For the night value, day weight was subtracted from the night weight (1800 - 0600). Values were recorded then assigned to physiological state, with early pregnancy encompassing d1-6, mid pregnancy d7-13 and late pregnancy d14-parturition, day of parturition, and then early lactation d1-4, mid lactation d5-8 and late lactation d9-12. Kcal of intake was determined by multiplying grams consumed by 3.85 kcal/g for CON, or 5.24 kcal/g for HF.

Fecal collection, extraction and analysis of corticosterone content

Feces was collected twice daily from the cages to capture day (0600-1800) and night (1800-0600) output in early pregnancy (EP) d5-d7, mid pregnancy d7-d13, late pregnancy d14 to parturition (~d16), and lactation from day 4 to day 6. Mice were transferred to a new cage with fresh corn cob bedding and fecal material was separated from the bedding, collected, and stored at -20˚C.

To extract corticosterone (pg.mL^{-1}), feces were aliquoted to ~0.2g and then ground using a Precellys 24 Lysis & Homogenization (Bertin Technologies, Montigny-le-Bretonneux, France) at Purdue University's Bindley Life Sciences. After grinding, 2 mL/0.2 g of ethanol was added, and the sample was centrifuged.

Approximately 1.3mL of the supernatant was pipetted into 2 separate tubes and dried in the speed vac for an hour. Once samples were dried, they were stored at -20˚C. Samples were reconstituted in 32.5µL of absolute ethanol and analyzed using the corticosterone Enzyme Linked Immunosorbent Assay (ELISA) from Arbor Assays (catalog no. K014, Ann Arbor, MI, USA) following manufacturer's protocol. Absorbance was read at 450 nm on the Spark 10M multimode microplate reader (Tecan Trading AG, Switzerland).

Lipid profiling analysis

Lipid extraction was performed according to the Bligh & Dyer protocol [28]. Briefly, 10 µL of milk and 100 µL of plasma were used for extraction, and deionized water (DI H_2O) was added in a microtube to bring the final volume to 200 µL. Tissue samples were individually weighed and ~126 mg of liver and ~156 mg of mammary gland was homogenized in 2 mL vials with 1.4 mm ceramic (zirconium oxide) beads with 500 µL of water using the Precellys24 tissue homogenizer (Bertin Technologies, Rockville, MD, USA). Part of the homogenate (200 µL) was transferred to a microtube. Then, for all the matrices (liver, mammary gland, milk and plasma), 550 µL of methanol and 250 µL of chloroform ($CHCl_3$ + 0.01% BHT) were added and vortexed for 10 seconds to form a one-phase solution. The samples were incubated for 15 min at 4 °C. Next, another 250 µL of ultrapure H_2O and 250 µL of $CHCl_3$ + 0.01% BHT were added. Samples were then centrifuged at 15,000 g for 10 min to enhance polar from nonpolar phase separation (two-phase solution). The organic phase was transferred to a new tube. Additionally, an aliquot of the lipid extract of each sample was mixed by treatment to produce pooled samples. All tubes were dried in a centrifugal evaporator (Savant SpeedVac AES2010, ThermoFisher Scientific, San Jose, CA). Dried lipid extracts were stored at −80 °C until mass spectrometry analysis.

MRM profiling analysis was conducted in two phases, discovery and screening. For the discovery phase, samples pooled by treatment and tissue-matrix (16 pools) were profiled for a list of MRMs related to lipid species generated by combining the *m/z* for the molecular ion based on the LIPID MAPS® online database and a class- or fatty acyl loss-specific product ion [29]. To prepare such list, constitutional isomers were combined into one single entry in which only the lipid class, number of total carbons across fatty acyl residues and unsaturation number is listed. This strategy allowed compaction of the large number of entries (16,257) by one order of magnitude (3,437 MRMs in the final list) due to the large number of isobaric lipids. The 3,437 MRMs were profiled in the discovery step for the pooled samples of each experimental group by tissue-matrix. Of the 3,437 MRM profiled in the discovery phase, 1,491 were found to be at least 1.3-fold higher than the blank in at least one pooled sample. These 1,491 MRM were then used to interrogate individual samples in the screening phase of the MRM profiling analysis.

Due to the limited time of signal provided by the sample at flow injection, the 1,491 MRMs profiled in the screening phase, were organized into 10 methods (Supplementary file S1), each one involving 2 minutes of

data acquisition for a maximum of 250 MRM transitions. Dried lipid extracts were reconstituted in acetronitrile (ACN) + methanol (MeOH) + 300 mM ammonium acetate (NH_4Ac) 3:6.65:0.35 (v/v), with the same volume dried, which was 300 µL for pools, and 200 µL for each sample, composing the stock solution. The reconstituted extracts were individually diluted 100X for milk, 400X for mammary, 600X for liver and 250X for plasma, with injection solvent ACN + MeOH + 300 mM NH_4Ac 3:6.65:0.35 (v/v). The screening phase of the MRM profiling was conducted by injecting 8 µL of the diluted lipid extract to the ESI source of an Agilent 6410 QQQ mass spectrometer (Agilent Technologies, Santa Clara, CA, USA) using a micro-autosampler (G1377A). The precursor ion selection window was 0.7 Th. The capillary pump connected to the autosampler operated with a flow of 10 µL/min and a pressure of 150 bar. Capillary voltage on the instrument was 3.5-5 kV and the gas flow was 5.1 L/min at 300°C.

The ion intensities of all MRMs were recovered, and each lipid ion was normalized by the total ion intensity of each sample (normalization by SUM) within each lipid class (phosphatidylcholines (PC), phosphatidylethanolamines (PE), phosphatidylserines (PS), phosphatidylinositol (PI), phosphatidylglycerol (PG), diacylglycerols (DG), triacylglycerols (TG), ceramides (Cer), sphingomyelins (SM), cholesteryl ester (CE), acyl-carnitines (AC), and free fatty acids (FFA), to acquire relative amounts. Subsequently, all data were consolidated into a unified file for each matrix (liver, mammary, milk, and plasma). Data analysis was performed using MetaboAnalyst 6.0 [30]. Following upload, data were normalized by auto-scaling and checked for normal distribution. Multivariate statistical tests included principal component analysis (PCA), and linear model, and univariate tests included T-test, were used to identify lipids significantly influenced by diet and PS treatment.

Data and statistical analysis

Statistical analyses, other than lipidome data, were performed using SAS 9.4 (SAS Inst. Inc., Cary, NC). The data were analyzed in a completely randomized design, and the animal was considered an experimental unit. The animals were randomly distributed in 1 of 2 dietary treatments and subsequently females were moved into one of the two experimental light rooms. The normality of the residues was verified by the Shapiro-Wilk test (UNIVARIATE procedure of SAS), and the Levene test compared the homogeneity of the variances. Variables with a continuous distribution such as feed intake, dam weight, birth litter size, weigh suckle weigh, TG, protein, lactose, prolactin and corticosterone concentration were analyzed using the MIXED procedure of SAS. When the time factor was not present, the statistical model included treatments (diet and light), stage, period of the day (day or night) as fixed effects, and mice as random effects. When the time factor was present (dam weight and litter weight), repeated measures models were fitted by multiple mixed linear models using the MIXED procedure of SAS, in which the statistical model included: the fixed effects of treatments (diet and light), period/day and their interaction, and the random effect of the animal. The experimental unit was the mice. The SLICE option using the LSMEANS/PDIFF command was used to explore the interactions of data

collection. The Kenward-Roger method was used to correct the degree of freedom of the denominator for the F test. The time of day of birth and pup death were evaluated by Chi-Square Test. The covariance structure was determined based on the lowest Akaike (AIC) information criteria value. Significance level was set at $P < 0.05$ and tendency towards significance at $0.05 < P < 0.10$.

Results

Feed intake

Diet, light, period of the day, and physiological stage significantly influenced the number of grams of feed consumed by mice ($P<0.05$, Table 1 in Supplementary File S2). Feed intake, measured in kilocalories, was also significantly affected by light, time of day, stage, and the interactions between light and period of the day, as well as the three-way interaction between stage, light, and period of the day ($P<0.05$, Table 1 in Supplementary File S2). Overall, HF diet reduced feed intake in grams and the exposure to PS increased feed intake in both grams and kilocalories (Table 1 in Supplementary File S2). Peak kcal intake occurred during lactation (grams and kcal), with mid and late lactation showing the highest levels ($P<0.01$). Mice consumed more food during the night (grams and kcal) (Table 1 in Supplementary File S2).

There was a significant interaction ($P<0.01$, Table 1 in Supplementary File S2) between light treatment and time of day for diet consumed in grams (Fig 2A) and kcal (Fig 2B). Post-hoc analysis revealed that both LD and PS mice consumed the most food during the night period. However, comparing the daytime period, PS mice had a higher feed intake (grams and kcal) compared to LD mice (Fig 2A and 2B). There was also a tendency for a three-way interaction between stage, light, and period of the day to influence feed intake in grams ($P = 0.05$, Table 1 in Supplementary File S2), with several significant differences observed. Specifically, within each light treatment, there was a trend for PS mice to consume more feed during the night and day in mid and late lactation compared to any period of pregnancy (Fig 2C and 2D). Additionally, during the daytime, PS mice had a tendency for higher feed intake in the late stages of pregnancy and late lactation (Fig 2C and 2D). Considering the feed intake in kcal, mice exposed to PS treatment exhibited greater feed intake at night during mid and late lactation stages, also an increase in consumption during the day in late pregnancy and mid and late lactation (Fig 2C and 2D). Interestingly, mice exposed to PS had their second highest food intake during two specific periods: the nighttime period of early lactation and the daytime period of late lactation. The food intake was the same in both periods (Fig 2C and 2D).

Fig 2. Impact of light/dark (LD) exposure and phase shifts (PS) on diurnal feed intake (A, B) and effect of light exposure, physiological stage, and time of day on feed intake in mice (C, D). LD, regular cycles of 12 hours of light and 12 hours of dark, and PS, mice exposed to 6-hour shifts in light-dark cycles every three days. Values are LS means ± SEM. Fig A and B: different letters indicate significant differences at $P <0.001$, with

uppercase indicating a difference between treatments during the day, and lowercase indicating a difference between treatments during the night. Fig C and D: different letters indicate a tendency ($P = 0.05$) for feed intake in grams and a significant difference ($P < 0.05$) for feed intake in kcal, with uppercase indicating a difference between day and night for LD light treatment, and lowercase indicating a difference between day and night for PS light treatment.

Fecal corticosterone levels and fecal weight

Physiological stage had an overall effect on fecal corticosterone levels in mice ($P = 0.0003$, Table 2 in Supplementary File S2), with corticosterone levels markedly higher in late pregnancy compared to early pregnancy and lactation stages. However, a significant interaction between light and physiological stage was observed for corticosterone levels ($P = 0.004$, Fig 3A). Mice exposed to PS exhibited similar corticosterone levels across all stages, deviating from the typical pattern seen in the LD group, where corticosterone levels were highest in late pregnancy, followed by early pregnancy, and lowest during lactation (Fig 3A). Total fecal weight produced over a 24 h period was greater in the PS group compared to those in the LD group ($P = 0.001$, Table 2 in Supplementary File S2). Additionally, a significant interaction between diet and light exposure was observed ($P = 0.008$, Fig 3B), with mice exposed to PS and fed a HF diet producing the greatest amount of feces weight. Corticosterone output levels were significantly affected by both light exposure and physiological stage ($P<0.05$, Table 2 in Supplementary File S2). Levels were higher in the PS group compared to the LD group. Furthermore, there was a tendency ($P = 0.068$, Fig 3C) for an interaction between light exposure and period of the day, with PS treatment showing a near-significant increase in corticosterone levels at night compared to the day. Additionally, this trend suggests that corticosterone levels were higher at night under PS treatment compared to the LD treatment during the same period (Fig 3C).

Fig 3. Interaction between light and stage on fecal corticosterone levels (A), interaction between light exposure and diet on fecal weight (B) and interaction between light and day on corticosterone output in feces (C). LD, regular cycles of 12 hours of light and 12 hours of dark, and PS, mice exposed to 6-hour shifts in light-dark cycles every three days. Control (CON) mice were fed a diet of 10% fat, and high fat (HF) diet were fed a diet of 60% fat. Values are LS means ± SEM. Early pregnancy (EP) was from d5-d7, late pregnancy (LP) was from d14-d16, and lactation was d4-d6. Fig A: This interaction was significant, with a P-value of 0.004. Uppercase indicates a significant difference between stages within LD light, and lowercase indicates a significant difference between stages within PS light. Fig B: This interaction was significant, with a P-value of 0.008. Bracket indicates a significant difference ($P<0.002$) between diets within lights. Uppercase indicates a significant difference between CON diet, and lowercase indicates a significant difference between HF diet across the lights. Fig C: This interaction tended to be significant, with a P-value of 0.068. Uppercase indicates a

tendency to be a significant difference between day, and lowercase indicates a tendency to be a significant difference between night across the lights.

Dam's weight, prolactin, TG and HbA1c

Overall, dam weight was significantly increased by the HF diet (P = 0.041, Table 3 in Supplementary File S2). Additionally, dams were heavier during the lactation d12 compared to the pre-pregnancy period (P < 0.001, Table 3 in Supplementary File S2). A significant interaction between diet and stage was observed (P = 0.026, Fig 4). Post hoc analysis indicated after 4 weeks of diet feeding pre-pregnancy, mice fed a HF diet gained more weight than those on a CON diet. However, no significant differences in dam weight were observed by lactation d12. Neither PS treatment, nor diet impacted prolactin, TG or HbA1c in dam's plasma (P>0.05, Table 3 in Supplementary File S2).

Fig 4. Interaction of diet and physiological stage (pre-pregnancy and lactation d12) on dam's weight. Control (CON) mice were fed a diet of 10% fat, and high fat (HF) diet were fed a diet of 60% fat. Values are LS means ± SEM. Mice were fed respective diets *ad libitum* 4 weeks before mating. Neonates were euthanized on lactation day 12. Interaction diet*physiological stage had a P-value of 0.026. Brackets indicate significant difference at P <0.001 between diets within pre-pregnancy. Uppercase indicates a difference between CON diet, and lowercase indicating a difference between HF diet across the stages.

Nutritional Composition of Milk

Neither diet nor PS treatment impacted levels of TG and protein in milk (P>0.05, Table 4 in Supplementary File S2). Diet had an overall effect on lactose level, with the HF fed animals having greater milk lactose content (P = 0.007, Table 4 in Supplementary File S2). A significant interaction between diet and light was observed for milk lactose levels (P = 0.037, Fig 5). In the CON diet group, mice treated with PS light had lower milk lactose levels compared to those in the LD treatment. However, in the HF diet group, light treatment had no effect on milk lactose levels. Across diets, there was no difference in milk lactose concentration for mice exposed to LD light. However, mice exposed to PS light had higher milk lactose levels when fed the HF diet than CON diet.

Fig 5. Impact of diet and light on milk lactose. LD, regular cycles of 12 hours of light and 12 hours of dark, and PS, mice exposed to 6-hour shifts in light-dark cycles every three days. Control (CON) mice were fed a diet of 10% fat, and high fat (HF) diet were fed a diet of 60% fat. Values are LS means ± SEM. Interaction diet*light had a P-value of 0.037. Different letters indicate significant difference at P <0.001, with uppercase indicating a difference between LD, and lowercase indicating a difference between PS.

Litter weight, birth litter size, weight suckle weight, time of day of birth and pup survival to 24 h

Neither birth litter size nor milk production level on lactation day 10, measured using weigh-suckle-weigh (WSW), was affected by maternal diet nor PS exposure (P>0.05, Table 5 in Supplementary File S2). Pup survival to 24h was not affected by diet nor light treatment (P>0.05, Table 5 in Supplementary File S2). However, there was a tendency (P = 0.056, Fig 6A) for light treatment to influence the time of day of birth, but diet had no effect on time of day of birth (P = 0.407, Fig 6B). In the LD group, 70.6% of births occurred during the day, whereas in the PS group, only 37.5% of births occurred during the day.

Fig 6. Interaction between light and time of day (TOD) of birth (A) and interaction between diet and time of day (TOD) of birth (B). LD, regular cycles of 12 hours of light and 12 hours of dark, and PS, mice exposed to 6-hour shifts in light-dark cycles every three days. Control (CON) mice were fed a diet of 10% fat, and high fat (HF) diet were fed a diet of 60% fat. Values are LS means ± SEM. Significance was determined using a Chi-square test at a P <0.05. Interaction light*time of day of birth had a P-value of 0.056 and diet*time had a P-value of 0.407.

Litter weight was significantly affected by diet (P<0.001, Table 5 in Supplementary File S2). By lactation d12, the mean litter weight across all treatments was 74.25 g, compared to 13.81 g on the day of birth (0d) (P<0.0001). This resulted in a significant interaction (P<0.0001) between diet and day with HF diet markedly increasing the litter growth rate from day 8 to day 12 (Fig 7).

Fig 7. Impact of phase shift (PS) and high fat (HF) diet on litter growth. LD, regular cycles of 12 hours of light and 12 hours of dark, and PS, mice exposed to 6-hour shifts in light-dark cycles every three days. Control (CON) mice were fed a diet of 10% fat, and high fat (HF) diet were fed a diet of 60% fat. Values are LS means ± SEM.

Lipid profiles of Liver, Mammary, Milk and Plasma

Scores plots of principal component analysis (PCA) demonstrate that diet, but not light treatment, had a profound effect on lipid profiles of the liver, mammary gland, milk and plasma (Fig 8).

Fig 8. PCA of lipid profiles in liver, mammary gland, milk, and plasma affected by diet but not by light. Red and green represent CON and HF diet, respectively, while triangles and circles represent the PS and LD treatment, respectively.

Linear model analysis of the overall effect of diet revealed that among 1204 lipids detected in the screening phase, diet altered 67.1% in milk, 58.1% in mammary gland, 27.2% in the liver, and 10.9% in plasma at P-adj<0.05 (Table 1).

Table 1. Percentage of lipids affected by diet in liver, mammary gland, milk, and plasma based on linear model analysis

	Liver		Mammary		Milk		Plasma	
	**n**	**%**	**n**	**%**	**n**	**%**	**n**	**%**
Different at P-adj<0.05	328	**27.2**	700	**58.1**	808	**67.1**	131	**10.9**
Not different	876	72.8	504	41.9	396	32.9	1073	89.1
Total	1204	100	1204	100	1204	100	1204	100

Across all tissues-matrices HF diet had the greatest impact on TG class of lipids, both increasing and decreasing abundance of individual species (Table 2). In liver, mammary and milk HF diet increased a relatively large proportion diacylglycerol (DG), whereas in the plasma the distribution of this class of lipids was relatively unchanged. In the liver the relative distribution of 15.05% of PC, 9.22% of PE and 7.28% PI lipids were decreased by HF diet. In the mammary gland 20.45% of PC lipids were decreased by HF, and 16.96% of PC and 15.90% of PE were decreased in milk. In plasma, 32.47% of PC lipid species were decreased (Table 2).

Table 2. Distribution of lipid classes affected by diet in liver, mammary gland, milk, and plasma of mice fed with HF or CON diet according to linear model analysis

	Liver				Mammary gland				Milk				Plasma			
	HF>CON		CON>HF		HF>CON		CON>HF		HF>CON		CON>HF		HF>CON		CON>HF	
	n	%	n	%	n	%	n	%	n	%	n	%	n	%	n	%
TG	51	**41.80**	109	**52.91**	243	**50.63**	83	**37.73**	283	**53.90**	107	**37.81**	26	**48.15**	13	**16.88**
CE	3	2.46	2	0.97	2	0.42	2	0.91	2	**0.38**	17	6.01	0	**0.00**	4	5.19
Cer	4	3.28	1	0.49	4	0.83	2	0.91	1	0.19	0	0.00	0	0.00	1	1.30
SM	8	6.56	11	5.34	3	**0.63**	12	5.45	1	**0.19**	15	5.30	9	16.67	9	**11.69**
DG	39	**31.97**	12	5.83	165	**34.38**	22	**10.00**	181	**34.48**	14	4.95	0	**0.00**	4	5.19
PC	7	**5.74**	31	**15.05**	9	**1.88**	45	**20.45**	5	**0.95**	48	**16.96**	8	**14.81**	25	**32.47**
PE	5	**4.10**	19	**9.22**	6	**1.25**	17	**7.73**	2	**0.38**	45	**15.90**	8	**14.81**	6	**7.79**
PI	1	**0.82**	15	**7.28**	4	**0.83**	9	4.09	4	**0.76**	10	3.53	2	**3.70**	15	19.48
PS	2	1.64	5	2.43	1	**0.21**	13	5.91	3	**0.57**	14	4.95	1	1.85	0	0.00
PG	0	0.00	0	0.00	42	**8.75**	10	4.55	42	**8.00**	9	3.18	0	0.00	0	0.00
CAR	0	0.00	0	0.00	1	0.21	2	0.91	1	0.19	3	1.06	0	0.00	0	0.00
FFA	2	1.64	1	0.49	0	0.00	3	1.36	0	0.00	1	0.35	0	0.00	0	0.00
Total	122	100	206	100	480	100	220	100	525	100	283	100	54	100	77	100

The impact of diet on total carbon length and unsaturation across the fatty acyl groups within each lipid class was also analyzed. HF diet increased the percentage of DG in liver with total carbons across the two-fatty acyl ≤ 31 and between 37 to 40 and increased percent of DG with higher degree of unsaturation compared to those in the CON group (Fig 9).

In the mammary gland, mice fed HF diet exhibited a higher proportion of TG lipid class with longer carbon chains, ranging from 50 to 59. Additionally, there was a notable increase in the degree of unsaturation within these lipids, compared to the CON mice (Fig 9). HF diet also shifted distribution of DG in the mammary gland, with a greater proportion of 34 to 40 carbons, in contrast to the 37 to 44 carbon range observed in the CON diet group (Fig 9). Despite this alteration, the pattern of unsaturation of DG lipids in the mammary gland, remained largely similar to CON diet, although with a reduction in proportion and a slight increase noted for unsaturation of 3 to 5, 10, and 11.

Diet influenced milk TG total carbon number length across fatty acyl groups. Chains primarily ranged from 42 to 48 carbons in milk of mice fed CON diet, and in milk of mice on HF diet total carbon length were primarily greater than 50 (Fig 9). Diet also altered the distribution of milk TG by total number of unsaturated bonds, transitioning from a predominance of 0 to 4 unsaturation in the CON diet to 6 to 8 in milk of mice fed HF diet. Diacylglycerols comprised 34.48% of the total lipids altered in milk by HF diet (Table 2). Analysis revealed a U-shape distribution of DGs with a greater relative abundance in the HF group, with predominant degrees of unsaturation being 1, 2, 3, 8, and 9, and chain lengths ranging from 24 to 40 carbons (Fig 9). In contrast, DGs in CON groups displayed a different profile, characterized by a mix of saturated and unsaturated forms, with unsaturation levels primarily at 0, 1, 7, and 8, and longer chain lengths of 41 to 44 carbons.

Diet significantly impacted the TG composition in the plasma of mice, with those on the HF diet exhibiting longer carbon chains, ranging from 53 to 57 carbons across the three fatty acyl groups, and a higher prevalence of unsaturated bonds, with 6 to 8 double bonds (Fig 9). In contrast, TG of CON fed mice had shorter carbon chains, ranging from 48 to 53 carbons, and a different unsaturation profile, predominantly featuring 1, 2, and 9 double bonds. In plasma from mice fed the HF diet, a greater number of the total PC species profiled were significantly reduced compared to CON group. The PC species more abundant in plasma of HF mice had carbon chains ranging from 35 to 38 carbons and number of unsaturated bonds were 1, 2, and 4 across the two acyl molecules (Fig 9). In contrast, PC species more abundant in the CON group had carbon chains of 20 and 38 carbons, with 0 and 1 unsaturated bonds.

Fig 9. Effects of HF diet on distribution of lipid classes in the liver, mammary gland, milk and plasma by total carbon chain length and degree of unsaturation

Linear regression analysis indicated no significant effect of light treatment on lipid profiles across all samples. Therefore, we tested whether within each diet lipid profiles differed between LD and PS treated mice using t-test. Differences in relative abundance of lipids between LD and PS mice became apparent only after reducing the stringency to raw P-value < 0.05 (Table 3). Within CON diet, PS treatment significantly altered the abundance of 6.5%, 3.2%, 2.9%, and 0.6% of lipids profiled in plasma, mammary gland, liver and milk (Table 3). Within the HF diet, 11.7%, 1.9%, 0.9%, and 0.9% in mammary gland, plasma, liver and milk, respectively.

Table 3. Comparison of significant lipid changes in various matrices under different light treatments within HF and CON diets

	CON LD vs. PS				HF LD VS. PS			
	**Liver**	**Mammary**	**Milk**	**Plasma**	**Liver**	**Mammary**	**Milk**	**Plasma**
FDR P<0.05	0	0	0	0	0	0	0	0
Raw P<0.05	35 (2.9%)	33 (3.2%)	6 (0.6%)	67 (6.5%)	11 (0.9%)	120 (11.7%)	9 (0.9%)	19 (1.9%)
Total	1204 (100%)	1204 (100%)	1204 (100%)	1204 (100%)	1204 (100%)	1204 (100%)	1204 (100%)	1204 (100%)

One-factor analysis, T-test with raw P-value 0<0.05. Significant lipid species are described in supplementary file S3.

No patterns in lipids modified by light treatment within the CON diet group were observed for any tissue-matrices (Supplementary File S4). PS treatment in the HF diet group altered the relative distribution of 120 lipids in mammary gland, of which 95% were decreased in abundance, whereas, in the CON diet group, PS treatment affected 33 lipids, with 78.8% showing increased abundance. Of the lipids reduced by PS treatment within HF diet, 45 were PC and 32 PE (Fig 10A). In contrast, these same lipid classes were increased by PS with CON diet, which 7 were PC and 12 were PE (Fig 10B).

The response to PS treatment was clearly influenced by the HF diet, as different lipid classes were downregulated in one diet but upregulated in the other, demonstrating light-diet interactions based on relative amount. To identify the consistent impact of PS treatment regardless of the diet, we analyzed lipids, from the most abundant classes, that were downregulated in the HF-PS group and upregulated in the CON-PS group within the PC and PE classes using the Venny 2.1 tool [31]. This comparison explicitly shows the diet by light interaction (Fig 10C and 10D). For the PC class, we found four lipid species and their isobars that were consistently affected by PS treatment, regardless of the diet (Fig 10C and Table 4). Similarly, for the PE class, we identified eight lipids and their isobars that were similar under both diets (Fig 10D and Table 4).

Fig 10. Distribution of downregulated and upregulated lipids in mammary gland under PS light treatment within the HF or CON diet (A, B) and lipid classes downregulated in HF-PS and upregulated in CON-PS highlight the consistent effect of PS treatment across diets (C, D).

Table 4. Impact of PS treatment on lipid species and their isobars in the PC and PE classes across different diets

Tentative attribution	Parent ion (*m/z*)	Product ion (*m/z*)
PC(14:0), LPC(15:0), LPC(O-16:0)	482.4	184.1
PC(33:3), PC(O-34:3), PC(P-34:2)	742.6	184.1
PC(42:4)	866.7	184.1
PC(44:5)	892.7	184.1
PE(33:2), PE(O-34:2), PE(P-34:1)	702.5	561.5
PE(37:3), PE(O-38:3), PE(P-38:2)	756.6	615.6
PE(39:6), PE(O-40:6), PE(P-40:5)	778.6	637.6
PE(41:6), PE(O-42:6)	806.6	665.6
PE(42:10), PE(41:3), PE(P-42:2)	812.6	671.6
PE(42:5)	822.6	681.6
PE(42:8), PE(41:1), PE(O-42:1), PE(P-42:0)	816.7	675.7
PE(44:4)	852.6	711.6

Discussion

Maternal adaptations to pregnancy and lactation can be modified by the female's environment and nutrition, impacting the development of their offspring. Herein we described the impact of circadian disrupting environments and high-fat diet on maternal physiology-lipidome, milk composition and neonate outcomes. Our primary findings include alteration in maternal feed intake, fecal corticosterone and milk lactose levels by both diet and circadian disrupting environments. Within the CON diet group, milk lactose levels of PS mice decreased, while on a HF diet, milk lactose levels increased. Additionally, corticosterone levels were elevated for PS group across all physiological periods—early pregnancy, late pregnancy and lactation. HF diet increased litter growth during lactation and profoundly impacted the distribution of lipid profiles in mothers' liver, plasma, mammary and milk, increasing abundance of both longer and shorter chains and altering levels of double bonds. The shifts in lipid profiles induced by HF diet likely reflect the greater dependence on lipids from diet to support energetic needs of dam and milk fat production as well as potentially oxidative damage. Whereas PS treatment had no overall effect on lipid profiles, there was diet by light treatment interactions on the PC and PE lipids in the mammary gland. The PS treatment tended to increase the number of births during the night hours. Moreover, HF diet consumption significantly decreased overall feed intake (grams) and increased fecal weight, and dam weight.

The reduction in feed intake in grams by the HF diet was expected and agrees with our previous study that found the same changes in eating behavior [32], indicating less of a need of mice to eat to support pregnancy and lactation when given an energy dense diet. The HF diet increased lactose levels in the milk of dams. This finding is consistent with our previous report [33] and may reflect a more concentrated milk (lower percent of

water). However, lack of an effect of diet on milk protein or TG concentration does not support this interpretation. Alternatively, the HF diet may have provided greater substrates for lactose synthesis. The HF diet supplies more glycerol, a primary substrate for gluconeogenesis. In the mammary gland, glucose derived from gluconeogenesis is converted to galactose, which is then used for lactose synthesis [33–36]. Glycerol may have also been sourced from the greater energy stores of the HF mice, which weighed more than CON mice at the onset of pregnancy. These HF mice appeared to mobilize their fat stores to support energetic needs throughout the experiment, as evident by the lack of weight difference between the diet groups at the end of the study [32].

In addition to the overall effect of HF diet on elevating milk lactose, the significant diet by light interaction indicated that within the CON group the PS treatment significantly reduced milk lactose concentration. Circadian rhythms play a role in regulating milk production, and our group has previously shown that continuous light exposure, which disrupts circadian clocks, decreased lactose synthesis [32]. Our previous studies also showed a circadian rhythm of LALBA expression, a key component of the lactose synthase enzyme [37]. Work of others demonstrated circadian rhythms of lactose synthesis [38–40]. Therefore, circadian disrupting environments may depress milk lactose content. If these findings translate to humans, decreased lactose content of breast milk may negatively impact infants, as the lactose content of milk functions to promote neurological development of neonates and establishment of a healthy microbiome [41].

The higher fecal weight observed in the interaction of the HF diet and PS treatment may be due to the increased feed intake caused by circadian disruption. Mice in the PS group exhibited higher feed intake during the daytime, indicating a shift in feeding patterns due to circadian rhythm disruption. Although weight gain due to disrupted feeding patterns in the PS group was anticipated, it was only observed in the HF group. Similar to our previous study [32], the dams on the HF diet gained more weight, in the pre-pregnancy phase. The high caloric density of the HF diet led to increased body weight and fat accumulation. Additionally, hormonal changes involving corticosterone, leptin and insulin can influence energy balance and promote weight gain [42,43]. Obesity in females can negatively impact fertility by disrupting hormonal balance, impairing ovulation, and altering reproductive function leading to suboptimal outcomes [44–46]. Although, there was no effect of diet on fecal corticosterone concentration or total output, our previous studies found the HF diet increased fecal corticosterone output and hair corticosterone levels, both at the end of the pre-pregnancy period and on lactation day 12 [21,47 under review].

While we did not observe changes in milk production levels at the single weigh-suckle-weigh experiment on lactation day 10, the increased lactose concentration in milk of the HF group may have contributed to the higher litter weight, as lactose serves as a vital energy source for neonates supplying a substantial portion of the calories required for growth and development [48]. Although not different in this study, our previous study found a higher percent fat in milk of HF fed dams [33]. The HF milk as a calorie-dense food has the potential to increase growth rate of litters as well as provide bioactive lipids that support and optimize litter development [49].

The PS treatment disrupted the normal corticosterone fluctuations associated with the physiological transitions of the dam from early to late pregnancy and into lactation. Mice exposed to a 12-hour LD cycle showed changes in fecal corticosterone concentration between these stages, reflecting the hormonal adjustments necessary for impending parturition [50]. During pregnancy, elevated levels of estrogen and progesterone influence the hypothalamic-pituitary-adrenal (HPA) axis, resulting in an increase of corticosterone production [50–52]. Typically, there is a suppression of the HPA axis during lactation period to mitigate stress responses that could interfere with maternal behaviors, thereby protecting the offspring from elevated stress hormone levels in the milk and leading to lower corticosterone levels [53–57]. Such changes are essential for the healthy development of the offspring [58]. However, dams exposed to constant shifts in the photoperiod due to PS treatment exhibited persistently high corticosterone concentrations, which remained unchanged across the reproductive phases. This pattern potentially indicates a chronic stress response, as constant circadian or ultradian rhythm disruption can dysregulate the HPA axis. Consequently, this dysregulation results in sustained elevated corticosterone levels, through elevated glucocorticoids, which can impair the negative feedback mechanisms that typically regulate the HPA axis or increase adrenal sensitivity to adrenocorticotropic hormone (ACTH) [59–61]. Such alterations in corticosterone rhythms may disrupt metabolic processes, increase the risk of metabolic disorders, suppress immune function, and negatively impact various aspects of reproduction and maternal care [59,60,62]. Additionally, these disruptions can have long-term effects on offspring development and behavior [21,32,62].

HF diets have been shown to elevate oxidative stress and lipid peroxidation, resulting in higher MDA levels in the milk of HF-fed mice [32]. Oxidative stress is induced by high fat diets in rodent models, including during pregnancy and lactation [63–65]. A metanalysis of rodent studies found higher MDA levels in offspring of HF diet groups, and increased ROS and lower superoxide dismutase and catalase in offspring of mothers exposed to high-fat diet during pregnancy and lactation [66]. Thus, we explored lipidome profiles of animals for evidence of oxidative damage, hypothesizing there would be shifts in carbon lengths and double bonds, with HF fed animals to have both shorter and longer carbon chain lengths, and reduced amount of unsaturation.

Although oxidation can sometimes increase unsaturation by forming new double bonds, it typically cleaves fatty acid chains at carbon-carbon double bonds, resulting in shorter-chain fatty acids, fewer double bonds, and sometimes odd number of carbons [67–69]. The HF diet increased the number of carbons in TG found in the plasma, mammary gland, and milk, as well as in the DG of the liver and PC of the plasma. In contrast, animals on CON diet had a greater prevalence of DG with a higher number of carbons across fatty acyl group in the mammary gland and milk. These results suggest that the HF diet suppress *de novo* lipogenesis, while simultaneously increasing desaturation, which alter the lipid profile by enhancing the incorporation of fatty acids into different types of lipids in the body, thereby influencing the overall lipid composition and metabolism [70]. Although the expected outcome is the suppression of both de novo lipogenesis and desaturation, and

maintains or even increases the elongation of fatty acids [71], the observed increase in desaturation may occur in response to the abundance of dietary fat, which provides more substrates for desaturation enzymes.

Relative to animals on CON diet, HF diet increased fatty acid (FA) length in the TG lipid class but decreased length in the DG lipid class in the mammary gland and milk. The mammary gland primarily synthesizes fatty acids *de novo* that are less than or equal to 16 carbons, resulting in shorter FA chains incorporated into TG due to the high affinity of diacylglycerol acyltransferase 1 (DGAT1) for these substrates [72,73]. DG are intermediates to TG and phospholipid synthesis [74], with the metabolic channeling in the mammary gland primarily directing longer-chain DGs toward phospholipid synthesis, essential for the milk fat globule membrane structure [72]. HF diet can lead to lipid remodeling in mammary gland, including elongation and desaturation of fatty acids (FA) through enzymes such as stearoyl-CoA desaturase (SCD1) and elongases [75,76]. Elongation of FA (C16) to very-long-chain FA (C18 or longer) occurs via elongation of very long chain fatty acids (ELOVL)-mediated chain elongation [76,77]. Other studies have shown that the HF diet induces ELOVL expression [78] or enhances the elongation flux from C16 to C18:1 [79]. These changes in FA distribution in TG and DG in response to HF diet, as indicated by total carbon length and unsaturation, likely reflects the metabolic intermediates and final products.

Changing the FA chain lengths in TG and DG in milk can have significant consequences for neonates, potentially predisposing them to metabolic disorders or obesity later in life [80]. Longer-chain FA in TG increase the energy density of milk, leading to higher caloric intake, faster weight gain, and potentially long-term effects on body composition and metabolic health [81,82]. In contrast, shorter-chain FA in DG are more easily digested, whereas longer-chain FA in TG reduce fat absorption efficiency, potentially causing gastrointestinal discomfort or malabsorption issues [83]. Neonates, especially those with fatty acid oxidation disorders, might experience an increased risk of metabolic decompensation and accumulation of toxic metabolites in mitochondria [84,85]. Additionally, longer-chain FA in TG reduce the availability of medium-chain FA for rapid ketogenesis, which can impact brain development, as ketones are vital for neonatal brains [86]. Alterations in FA chain lengths can also affect milk fat globule membrane structure, changing the bioavailability of membrane-associated nutrients and the stability of milk fat globules, which in turn affects digestion and absorption [87–89].

Exposure to continual light-dark phase-shifts is similar to experiencing chronic jet lag, which disrupts the circadian system [90]. This disruption impacts the molecular and physiological processes across the body causing a misalignment in the timing of biological functions that are usually synchronized with the light-dark cycle and profoundly affect metabolic homeostasis to include lipid and glucose metabolism [91–93]. In addition, disrupting the normal feeding-fasting cycle also affects lipid metabolism [94].

Our study revealed that lipid class PC and PE were impacted by the PS treatment, in a diet specific manner, reducing several species under HF diet and increasing under CON diet across all matrices. PC and PE are the major phospholipids in the membrane, comprising 45-55% and 15-25%, respectively [95]. Hepatic

phospholipids, including PC and PE, decrease under HF diet conditions in mice with non-alcoholic fatty liver disease (NAFLD), a condition associated with high oxidative stress and liver damage [96]. In the mammary gland, the biosynthesis of PC and PE involves the CDP-choline (Kennedy) pathway and the phosphatidylethanolamine N-methyltransferase (PEMT) pathway [72,97]. Enzymes such as diacylglycerol choline/ethanolamine phosphotransferase (CEPT) and choline kinase α (CKα), all crucial in the Kennedy pathway, are influenced by diet, and hormonal changes during lactation, and possibly circadian rhythms [72,97,98]. Disruptions in these pathways, potentially through light interventions like PS treatment, may influence the nutrient composition of milk, and impact pup growth and development [87,95,98].

The PS treatment affected four lipids in the PC class and eight in the PE class, regardless of the diet. Notably, some lipids contained odd-chain fatty acids (OCFA) and ether lipids. OCFA are less common in mammals and form through processes like the use of propionyl-CoA, or obtained through dietary sources [95] or alpha-oxidation of even-chain fatty acids [99]. The observed reduction in ether lipids, particularly those with longer carbon chains, suggests an increase in oxidative stress, as ether lipids play a crucial role in resisting lipid peroxidation and scavenging reactive oxygen species (ROS) [100,101]. This heightened oxidative stress can damage cells, negatively impacting milk production and quality, and potentially leading to impaired pup growth, weakened immune systems, and hindered cognitive development [102–104]. These findings imply that PS light exposure might exacerbate oxidative stress in the mammary gland under HF diet conditions, highlighting the importance of lipid composition in maternal and neonatal health that may influence long-term health outcomes.

Conclusion

Our investigation explored the complex interaction between high-fat diet and exposure to circadian-disrupting light-dark phase shifts on various physiological parameters in pregnant and lactating mice that may contribute to the negative observations in offspring development in response to their exposure. High-fat diet and circadian disrupting PS treatment both altered feeding behavior. Dams subjected to HF showed reduced feed intake compared to CON group, while gaining more weight pre-pregnancy and showing no significant difference in weight at the end of lactation. PS increased feed intake without changes in dam or litter weight, suggesting a decrease in metabolic efficiency. Maternal fecal corticosterone levels varied across early and late pregnancy and into lactation, however when dams were exposed to PS, corticosterone levels did not vary across physiological stage, remaining relatively elevated and indicating a greater exposure to stress throughout pregnancy and lactation. Lactose content of milk increased in response to HF diet, however in the CON group PS exposure decreased lactose content. Although birth litter size and pup survival did not differ by light treatments, there was a tendency for PS births to occur at night, indicating a possible light-induced variation and suggesting potential circadian influences on the timing of parturition. HF diet profoundly impacted lipid

profiles, with alterations supporting evidence for a greater dependence on dietary fats and their elongation and desaturation as well as oxidative damage across all tissues and matrices. Lipidome profiles also indicated PS exposure may increase oxidative stress damage due to reducing PC and PE lipid classes levels as well as potentially reducing their ether lipids. The changes in maternal variables likely reflect her health and metabolic status and affect the nutritional and developmental environment of offspring, and findings here may provide insight into the impact of maternal nutrition and environment on fetal and neonate development.

References

1. Lunn RM, Blask DE, Coogan AN, Figueiro MG, Gorman MR, Hall JE, et al. Health consequences of electric lighting practices in the modern world: A report on the National Toxicology Program's workshop on shift work at night, artificial light at night, and circadian disruption. Sci Total Environ. 2017;607–608: 1073–1084. doi:10.1016/J.SCITOTENV.2017.07.056
2. Smolensky MH, Hermida RC, Reinberg A, Sackett-Lundeen L, Portaluppi F. Circadian disruption: New clinical perspective of disease pathology and basis for chronotherapeutic intervention. Chronobiol Int. 2016;33: 1101–1119. doi:10.1080/07420528.2016.1184678
3. Gangwisch JE. Epidemiological evidence for the links between sleep, circadian rhythms and metabolism. Obes Rev. 2009;10 Suppl 2: 37–45. doi:10.1111/J.1467-789X.2009.00663.X
4. Sen A, Sellix MT. The Circadian Timing System and Environmental Circadian Disruption: From Follicles to Fertility. Endocrinology. 2016;157: 3366–3373. doi:10.1210/EN.2016-1450
5. Rasmussen KM, Kjolhede CL. Maternal Obesity: A Problem for Both Mother and Child. Obesity. 2008;16: 929–931. doi:10.1038/OBY.2008.36
6. Casey T, Sun H, Burgess HJ, Crodian J, Dowden S, Cummings S, et al. Delayed Lactogenesis II is Associated With Lower Sleep Efficiency and Greater Variation in Nightly Sleep Duration in the Third Trimester. J Hum Lact. 2019;35: 713–724. doi:10.1177/0890334419830991
7. Casey TM, Plaut K, Boerman J. Circadian clocks and their role in lactation competence. Domest Anim Endocrinol. 2022;78: 106680. doi:10.1016/J.DOMANIEND.2021.106680
8. Casey TM, Plaut K. Circadian clocks and their integration with metabolic and reproductive systems: our current understanding and its application to the management of dairy cows. J Anim Sci. 2022;100. doi:10.1093/JAS/SKAC233
9. Evans MC, Anderson GM. Integration of Circadian and Metabolic Control of Reproductive Function. Endocrinology. 2018;159: 3661–3673. doi:10.1210/EN.2018-00691
10. Sen A, Hoffmann HM. Role of core circadian clock genes in hormone release and target tissue sensitivity in the reproductive axis. Mol Cell Endocrinol. 2020;501. doi:10.1016/J.MCE.2019.110655
11. Erren TC, Reiter RJ. Defining chronodisruption. J Pineal Res. 2009;46: 245–247. doi:10.1111/J.1600-

079X.2009.00665.X

12. Varcoe TJ, Boden MJ, Voultsios A, Salkeld MD, Rattanatray L, Kennaway DJ. Characterisation of the maternal response to chronic phase shifts during gestation in the rat: implications for fetal metabolic programming. PLoS One. 2013;8. doi:10.1371/JOURNAL.PONE.0053800

13. Gatford KL, Kennaway DJ, Liu H, Kleemann DO, Kuchel TR, Varcoe TJ. Simulated shift work disrupts maternal circadian rhythms and metabolism, and increases gestation length in sheep. J Physiol. 2019;597: 1889–1904. doi:10.1113/JP277186

14. Varcoe TJ, Wight N, Voultsios A, Salkeld MD, Kennaway DJ. Chronic phase shifts of the photoperiod throughout pregnancy programs glucose intolerance and insulin resistance in the rat. PLoS One. 2011;6. doi:10.1371/JOURNAL.PONE.0018504

15. Smarr BL, Grant AD, Perez L, Zucker I, Kriegsfeld LJ. Maternal and Early-Life Circadian Disruption Have Long-Lasting Negative Consequences on Offspring Development and Adult Behavior in Mice. Sci Reports 2017 71. 2017;7: 1–12. doi:10.1038/s41598-017-03406-4

16. Young BE, Johnson SL, Krebs NF. Biological Determinants Linking Infant Weight Gain and Child Obesity: Current Knowledge and Future Directions. Adv Nutr. 2012;3: 675. doi:10.3945/AN.112.002238

17. Sun B, Purcell RH, Terrillion CE, Yan J, Moran TH, Tamashiro KLK. Maternal high-fat diet during gestation or suckling differentially affects offspring leptin sensitivity and obesity. Diabetes. 2012;61: 2833–2841. doi:10.2337/DB11-0957/-/DC1

18. Samuelsson AM, Matthews PA, Argenton M, Christie MR, McConnell JM, Jansen EHJM, et al. Diet-induced obesity in female mice leads to offspring hyperphagia, adiposity, hypertension, and insulin resistance: a novel murine model of developmental programming. Hypertens (Dallas, Tex 1979). 2008;51: 383–392. doi:10.1161/HYPERTENSIONAHA.107.101477

19. Elahi MM, Cagampang FR, Mukhtar D, Anthony FW, Ohri SK, Hanson MA. Long-term maternal high-fat feeding from weaning through pregnancy and lactation predisposes offspring to hypertension, raised plasma lipids and fatty liver in mice. Br J Nutr. 2009;102: 514–519. doi:10.1017/S000711450820749X

20. De Paula Simino LA, De Fante T, Figueiredo Fontana M, Oliveira Borges F, Torsoni MA, Milanski M, et al. Lipid overload during gestation and lactation can independently alter lipid homeostasis in offspring and promote metabolic impairment after new challenge to high-fat diet. Nutr Metab (Lond). 2017;14. doi:10.1186/S12986-017-0168-4

21. Teeple K, Rajput P, Gonzalez M, Han-Hallett Y, Fernández-Juricic E, Casey T. High fat diet induces obesity, alters eating pattern and disrupts corticosterone circadian rhythms in female ICR mice. PLoS One. 2023;18: e0279209. doi:10.1371/JOURNAL.PONE.0279209

22. Li X, Liu X, Meng Q, Wu X, Bing X, Guo N, et al. Circadian clock disruptions link oxidative stress and systemic inflammation to metabolic syndrome in obstructive sleep apnea patients. Front Physiol. 2022;13: 932596. doi:10.3389/FPHYS.2022.932596/BIBTEX

23. LLabre JE, Trujillo R, Sroga GE, Figueiro MG, Vashishth D. Circadian rhythm disruption with high-fat diet impairs glycemic control and bone quality. FASEB J. 2021;35: e21786. doi:10.1096/FJ.202100610RR
24. Tortoriello D V., McMinn J, Chua SC. Dietary-induced obesity and hypothalamic infertility in female DBA/2J mice. Endocrinology. 2004;145: 1238–1247. doi:10.1210/EN.2003-1406
25. Feng W, Liu S, Zhang C, Xia Q, Yu T, Zhu D. Comparison of cerebral and cutaneous microvascular dysfunction with the development of type 1 diabetes. Theranostics. 2019;9: 5854. doi:10.7150/THNO.33738
26. Han BG, Hao CM, Tchekneva EE, Wang YY, Chieh AL, Ebrahim B, et al. Markers of glycemic control in the mouse: comparisons of 6-h- and overnight-fasted blood glucoses to Hb A1c. Am J Physiol - Endocrinol Metab. 2008;295: E981. doi:10.1152/AJPENDO.90283.2008
27. Dan K, Fujita H, Seto Y, Kato R. Relation between stable glycated hemoglobin A1C and plasma glucose levels in diabetes-model mice. Exp Anim. 1997;46: 135–140. doi:10.1538/EXPANIM.46.135
28. BLIGH EG, DYER WJ. A rapid method of total lipid extraction and purification. Can J Biochem Physiol. 1959;37: 911–917. doi:10.1139/O59-099
29. Conroy MJ, Andrews RM, Andrews S, Cockayne L, Dennis EA, Fahy E, et al. LIPID MAPS: update to databases and tools for the lipidomics community. Nucleic Acids Res. 2024;52: D1677–D1682. doi:10.1093/NAR/GKAD896
30. Pang Z, Lu Y, an Zhou G, Hui F, Xu L, Viau C, et al. MetaboAnalyst 6.0: towards a unified platform for metabolomics data processing, analysis and interpretation. Nucleic Acids Res. 2024;2024: 1–9. doi:10.1093/NAR/GKAE253
31. Venny 2.1.0. [cited 24 Jul 2024]. Available: https://bioinfogp.cnb.csic.es/tools/venny/
32. Teeple K, Rajput P, Scinto S, Schoonmaker J, Davis C, Dinn M, et al. Impact of high-fat diet and exposure to constant light on reproductive competence of female ICR mice. Biol Open. 2023;12. doi:10.1242/BIO.060088
33. Chen Y, Wang J, Yang S, Utturkar S, Crodian J, Cummings S, et al. Effect of high-fat diet on secreted milk transcriptome in midlactation mice. Physiol Genomics. 2017;49: 747–762. doi:10.1152/PHYSIOLGENOMICS.00080.2017
34. Sadovnikova A, Garcia SC, Hovey RC. A Comparative Review of the Cell Biology, Biochemistry, and Genetics of Lactose Synthesis. J Mammary Gland Biol Neoplasia. 2021;26: 181. doi:10.1007/S10911-021-09490-7
35. Shah AM, Wondisford FE. Tracking the carbons supplying gluconeogenesis. J Biol Chem. 2020;295: 14419. doi:10.1074/JBC.REV120.012758
36. Barry JM. Conversion of Glucose to Galactose in the Mammary Gland. Nat 1952 1694308. 1952;169: 878–879. doi:10.1038/169878a0

37. Casey TM, Crodian J, Erickson E, Kuropatwinski KK, Gleiberman AS, Antoch MP. Tissue-specific changes in molecular clocks during the transition from pregnancy to lactation in mice. Biol Reprod. 2014;90. doi:10.1095/BIOLREPROD.113.116137
38. Barkova EN, Nazarenko E V., Zhdanova E V. Diurnal variations in qualitative composition of breast milk in women with iron deficiency. Bull Exp Biol Med. 2005;140: 394–396. doi:10.1007/S10517-005-0500-2/METRICS
39. Lubetzky R, Mimouni FB, Dollberg S, Salomon M, Mandel D. Consistent Circadian Variations in Creamatocrit over The First 7 Weeks of Lactation: A Longitudinal Study. https://home.liebertpub.com/bfm. 2007;2: 15–18. doi:10.1089/BFM.2006.0013
40. Villalpando S, del Prado-Manríquez M, Stafford J, Delgado G. Diurnal variations in the fatty acid composition of milk fat from marginally nourished women. Arch Med Res. 1995;26 Spec No: 139–143.
41. Grenov B, Briend A, Sangild PT, Thymann T, Rytter MH, Hother AL, et al. Undernourished Children and Milk Lactose. Food Nutr Bull. 2016;37: 85–99. doi:10.1177/0379572116629024
42. Myers MG, Leibel RL, Seeley RJ, Schwartz MW. Obesity and Leptin Resistance: Distinguishing Cause from Effect. Trends Endocrinol Metab. 2010;21: 643. doi:10.1016/J.TEM.2010.08.002
43. Dallman MF, Pecoraro N, Akana SF, La Fleur SE, Gomez F, Houshyar H, et al. Chronic stress and obesity: A new view of "comfort food." Proc Natl Acad Sci U S A. 2003;100: 11696. doi:10.1073/PNAS.1934666100
44. Silvestris E, Lovero D, Palmirotta R. Nutrition and female fertility: An interdependent correlation. Front Endocrinol (Lausanne). 2019;10: 451315. doi:10.3389/FENDO.2019.00346/BIBTEX
45. Dağ ZÖ, Dilbaz B. Impact of obesity on infertility in women. J Turkish Ger Gynecol Assoc. 2015;16: 111. doi:10.5152/JTGGA.2015.15232
46. Zheng L, Yang L, Guo Z, Yao N, Zhang S, Pu P. Obesity and its impact on female reproductive health: unraveling the connections. Front Endocrinol (Lausanne). 2023;14. doi:10.3389/FENDO.2023.1326546
47. Reis LG, Teeple K, Schoonmaker JL, Davis C, Scinto S, Schinckel A, et al. Constant light and high fat diet alter daily patterns of activity, feed intake and fecal corticosterone levels in pregnant and lactating female ICR mice. PLoS One. 2024.
48. Romero-Velarde E, Delgado-Franco D, García-Gutiérrez M, Gurrola-Díaz C, Larrosa-Haro A, Montijo-Barrios E, et al. The Importance of Lactose in the Human Diet: Outcomes of a Mexican Consensus Meeting. Nutrients. 2019;11. doi:10.3390/NU11112737
49. Pimentel L, Gomes A, Pintado M, Rodríguez-Alcalá LM. Isolation and Analysis of Phospholipids in Dairy Foods. J Anal Methods Chem. 2016;2016. doi:10.1155/2016/9827369
50. Mcmullin TS, Lowe ER, Bartels MJ, Marty MS. Dynamic Changes in Lipids and Proteins of Maternal, Fetal, and Pup Blood and Milk during Perinatal Development in CD and Wistar Rats. Toxicol Sci. 2008;105: 260–274. doi:10.1093/TOXSCI/KFN124

51. Sheng JA, Bales NJ, Myers SA, Bautista AI, Roueinfar M, Hale TM, et al. The Hypothalamic-Pituitary-Adrenal Axis: Development, Programming Actions of Hormones, and Maternal-Fetal Interactions. Front Behav Neurosci. 2021;14: 601939. doi:10.3389/FNBEH.2020.601939/BIBTEX

52. Brunton PJ, Russell JA, Douglas AJ. Adaptive Responses of the Maternal Hypothalamic-Pituitary-Adrenal Axis during Pregnancy and Lactation. J Neuroendocrinol. 2008;20: 764–776. doi:10.1111/J.1365-2826.2008.01735.X

53. Stern JM, Goldman L, Levine S. Pituitary-adrenal responsiveness during lactation in rats. Neuroendocrinology. 1973;12: 179–191. doi:10.1159/000122167

54. D. Neumann I, Wigger A, Liebsch G, Holsboer F, Landgraf R. Increased basal activity of the hypothalamo-pituitary-adrenal axis during pregnancy in rats bred for high anxiety-related behaviour. Psychoneuroendocrinology. 1998;23: 449–463. doi:10.1016/S0306-4530(98)00023-7

55. Lightman SL, Windle RJ, Wood SA, Kershaw YM, Shanks N, Ingram CD. Peripartum plasticity within the hypothalamo-pituitary-adrenal axis. Prog Brain Res. 2001;133: 111–129. doi:10.1016/S0079-6123(01)33009-1

56. Kammerer M, Adams D, von Castelberg B, Glover V. Pregnant women become insensitive to cold stress. BMC Pregnancy Childbirth. 2002;2. doi:10.1186/1471-2393-2-8

57. De Weerth C, Buitelaar JK. Physiological stress reactivity in human pregnancy--a review. Neurosci Biobehav Rev. 2005;29: 295–312. doi:10.1016/J.NEUBIOREV.2004.10.005

58. Slattery DA, Neumann ID. No stress please! Mechanisms of stress hyporesponsiveness of the maternal brain. J Physiol. 2008;586: 377. doi:10.1113/JPHYSIOL.2007.145896

59. Azmi NASM, Juliana N, Azmani S, Effendy NM, Abu IF, Teng NIMF, et al. Cortisol on Circadian Rhythm and Its Effect on Cardiovascular System. Int J Environ Res Public Health. 2021;18: 1–15. doi:10.3390/IJERPH18020676

60. O'Byrne NA, Yuen F, Butt WZ, Liu PY. Sleep and Circadian Regulation of Cortisol: A Short Review. Curr Opin Endocr Metab Res. 2021;18: 178. doi:10.1016/J.COEMR.2021.03.011

61. Den Boon FS, De Vries T, Baelde M, Joëls M, Karst H. Circadian and Ultradian Variations in Corticosterone Level Influence Functioning of the Male Mouse Basolateral Amygdala. Endocrinology. 2019;160: 791–802. doi:10.1210/EN.2018-00767

62. Wright KP, Drake AL, Frey DJ, Fleshner M, Desouza CA, Gronfier C, et al. Influence of Sleep Deprivation and Circadian Misalignment on Cortisol, Inflammatory Markers, and Cytokine Balance. Brain Behav Immun. 2015;47: 24. doi:10.1016/J.BBI.2015.01.004

63. Luo Z, Xu X, Zhao S, Sho T, Luo W, Zhang J, et al. Inclusion of microbe-derived antioxidant during pregnancy and lactation attenuates high-fat diet-induced hepatic oxidative stress, lipid disorders, and NLRP3 inflammasome in mother rats and offspring. Food Nutr Res. 2019;63. doi:10.29219/FNR.V63.3504

64. Hatanaka Y, Wada K, Kabuta T. Maternal high-fat diet leads to persistent synaptic instability in mouse offspring via oxidative stress during lactation. Neurochem Int. 2016;97: 99–108. doi:10.1016/J.NEUINT.2016.03.008
65. Emiliano AF, De Cavalho LCRM, Da Silva Cristino Cordeiro V, Da Costa CA, De Oliveira PBR, Queiroz EF, et al. Metabolic disorders and oxidative stress programming in offspring of rats fed a high-fat diet during lactation: effects of a vinifera grape skin (ACH09) extract. J Cardiovasc Pharmacol. 2011;58: 319–328. doi:10.1097/FJC.0B013E3182244A51
66. Moraes-Souza RQ, Vesentini G, Paula VG, Sinzato YK, Soares TS, Gelaleti RB, et al. Oxidative Stress Profile of Mothers and Their Offspring after Maternal Consumption of High-Fat Diet in Rodents: A Systematic Review and Meta-Analysis. Oxid Med Cell Longev. 2021;2021. doi:10.1155/2021/9073859
67. Ayala A, Muñoz MF, Argüelles S. Lipid Peroxidation: Production, Metabolism, and Signaling Mechanisms of Malondialdehyde and 4-Hydroxy-2-Nonenal. Oxid Med Cell Longev. 2014;2014. doi:10.1155/2014/360438
68. Tyurina YY, Tyurin VA, Anthonymuthu T, Amoscato AA, Sparvero LJ, Nesterova AM, et al. "Redox lipidomics technology: Looking for a needle in a haystack." Chem Phys Lipids. 2019;221: 93. doi:10.1016/J.CHEMPHYSLIP.2019.03.012
69. Kodali ST, Kauffman P, Kotha SR, Yenigalla A, Veeraraghavan R, Pannu SR, et al. Oxidative Lipidomics: Analysis of Oxidized Lipids and Lipid Peroxidation in Biological Systems with Relevance to Health and Disease. 2020; 61–92. doi:10.1007/978-3-030-47318-1_5
70. Ottestad I, Hassani S, Borge GI, Kohler A, Vogt G, Hyötyläinen T, et al. Fish oil supplementation alters the plasma lipidomic profile and increases long-chain PUFAs of phospholipids and triglycerides in healthy subjects. PLoS One. 2012;7. doi:10.1371/JOURNAL.PONE.0042550
71. Duarte JAG, Carvalho F, Pearson M, Horton JD, Browning JD, Jones JG, et al. A high-fat diet suppresses de novo lipogenesis and desaturation but not elongation and triglyceride synthesis in mice. J Lipid Res. 2014;55: 2541. doi:10.1194/JLR.M052308
72. Walter L, Narayana VK, Fry R, Logan A, Tull D, Leury B. Milk fat globule size development in the mammary epithelial cell: a potential role for ether phosphatidylethanolamine. Sci Rep. 2020;10: 12299. doi:10.1038/S41598-020-69036-5
73. Mohammad MA, Haymond MW. Regulation of lipid synthesis genes and milk fat production in human mammary epithelial cells during secretory activation. Am J Physiol - Endocrinol Metab. 2013;305: E700. doi:10.1152/AJPENDO.00052.2013
74. Yen CLE, Stone SJ, Koliwad S, Harris C, Farese R V. DGAT enzymes and triacylglycerol biosynthesis. J Lipid Res. 2008;49: 2283. doi:10.1194/JLR.R800018-JLR200
75. Flowers MT, Ntambi JM. Role of stearoyl-coenzyme A desaturase in regulating lipid metabolism. Curr Opin Lipidol. 2008;19: 248. doi:10.1097/MOL.0B013E3282F9B54D

76. Wang X, Yu H, Gao R, Liu M, Xie W. A comprehensive review of the family of very-long-chain fatty acid elongases: structure, function, and implications in physiology and pathology. Eur J Med Res. 2023;28: 532. doi:10.1186/S40001-023-01523-7
77. Deák F, Anderson RE, Fessler JL, Sherry DM. Novel Cellular Functions of Very Long Chain-Fatty Acids: Insight From ELOVL4 Mutations. Front Cell Neurosci. 2019;13. doi:10.3389/FNCEL.2019.00428
78. Kozawa S, Honda A, Kajiwara N, Takemoto Y, Nagase T, Nikami H, et al. Induction of peroxisomal lipid metabolism in mice fed a high-fat diet. Mol Med Rep. 2011;4: 1157–1162. doi:10.3892/MMR.2011.560
79. Oosterveer MH, van Dijk TH, Tietge UJF, Boer T, Havinga R, Stellaard F, et al. High fat feeding induces hepatic fatty acid elongation in mice. PLoS One. 2009;4. doi:10.1371/JOURNAL.PONE.0006066
80. Lean SC, Candia AA, Gulacsi E, Lee GCL, Sferruzzi-Perri AN. Obesogenic diet in mice compromises maternal metabolic physiology and lactation ability leading to reductions in neonatal viability. Acta Physiol (Oxf). 2022;236. doi:10.1111/APHA.13861
81. Hanus O, Samkova E, Křížova L, Hasoňova L, Kala R. Role of Fatty Acids in Milk Fat and the Influence of Selected Factors on Their Variability—A Review. Mol A J Synth Chem Nat Prod Chem. 2018;23. doi:10.3390/MOLECULES23071636
82. Hua MC, Su HM, Yao TC, Liao SL, Tsai MH, Su KW, et al. The association between human milk fatty acid composition in mothers with an elevated body mass index and infant growth changes. Clin Nutr. 2024;43: 203–210. doi:10.1016/j.clnu.2023.11.028
83. Den Besten G, Van Eunen K, Groen AK, Venema K, Reijngoud DJ, Bakker BM. The role of short-chain fatty acids in the interplay between diet, gut microbiota, and host energy metabolism. J Lipid Res. 2013;54: 2325. doi:10.1194/JLR.R036012
84. J. Lawrence Merritt I, Norris M, Kanungo S. Fatty acid oxidation disorders. Ann Transl Med. 2018;6: 181–183. doi:10.21037/ATM.2018.10.57
85. Marsden D, Bedrosian CL, Vockley J. Impact of newborn screening on the reported incidence and clinical outcomes associated with medium- and long-chain fatty acid oxidation disorders. Genet Med 2021 235. 2021;23: 816–829. doi:10.1038/s41436-020-01070-0
86. Jadhav HB, Annapure US. Triglycerides of medium-chain fatty acids: a concise review. J Food Sci Technol. 2023;60: 2143. doi:10.1007/S13197-022-05499-W
87. Chai C, Oh S, Imm JY. Roles of Milk Fat Globule Membrane on Fat Digestion and InfantNutrition. Food Sci Anim Resour. 2022;42: 351. doi:10.5851/KOSFA.2022.E11
88. Thum C, Roy NC, Everett DW, McNabb WC. Variation in milk fat globule size and composition: A source of bioactives for human health. Crit Rev Food Sci Nutr. 2023;63: 87–113. doi:10.1080/10408398.2021.1944049

89. Maheshwari A, Mantry H, Bagga N, Frydrysiak-Brzozowska A, Badarch J, Rahman MM. Milk Fat Globules: 2024 Updates. Newborn. 2024;3: 19–37. doi:10.5005/JP-JOURNALS-11002-0085

90. Suarez-Trujillo A, Wernert G, Sun H, Steckler TS, Huff K, Cummings S, et al. Exposure to chronic light-dark phase shifts during the prepartum nonlactating period attenuates circadian rhythms, decreases blood glucose, and increases milk yield in the subsequent lactation. J Dairy Sci. 2020;103: 2784–2799. doi:10.3168/JDS.2019-16980

91. Casey T, Suarez-Trujillo AM, McCabe C, Beckett L, Klopp R, Brito L, et al. Transcriptome analysis reveals disruption of circadian rhythms in late gestation dairy cows may increase risk for fatty liver and reduced mammary remodeling. Physiol Genomics. 2021;53: 441. doi:10.1152/PHYSIOLGENOMICS.00028.2021

92. Klopp RN, Ferreira CR, Casey TM, Boerman JP. Relationship of cow and calf circulating lipidomes with colostrum lipid composition and metabolic status of the cow. J Dairy Sci. 2022;105: 1768–1787. doi:10.3168/jds.2021-21008

93. Beckett LM, Donkin SS, Casey T. Circadian disruption decreases gluconeogenic flux in late-gestation, nonlactating dairy cows. JDS Commun. 2023;4: 412–416. doi:10.3168/JDSC.2022-0353

94. Chaix A, Zarrinpar A. The effects of time-restricted feeding on lipid metabolism and adiposity. Adipocyte. 2015;4: 319. doi:10.1080/21623945.2015.1025184

95. Cockcroft S. Mammalian lipids: structure, synthesis and function. Essays Biochem. 2021;65: 813. doi:10.1042/EBC20200067

96. Mouskeftara T, Deda O, Papadopoulos G, Chatzigeorgiou A, Gika H. Lipidomic Analysis of Liver and Adipose Tissue in a High-Fat Diet-Induced Non-Alcoholic Fatty Liver Disease Mice Model Reveals Alterations in Lipid Metabolism by Weight Loss and Aerobic Exercise. Molecules. 2024;29. doi:10.3390/MOLECULES29071494/S1

97. Li Z, Vance DE. Thematic Review Series: Glycerolipids. Phosphatidylcholine and choline homeostasis. J Lipid Res. 2008;49: 1187–1194. doi:10.1194/JLR.R700019-JLR200

98. Monjes NM, Wagner PM, Guido ME. “Disruption of the molecular clock severely affects lipid metabolism in a hepatocellular carcinoma cell model.” J Biol Chem. 2022;298: 102551. doi:10.1016/j.jbc.2022.102551

99. Rodwell V. Harper’s Illustrated Biochemistry. 31st ed. McGraw-Hill;

100. Pradas I, Jové M, Huynh K, Puig J, Ingles M, Borras C, et al. Exceptional human longevity is associated with a specific plasma phenotype of ether lipids. Redox Biol. 2019;21: 101127. doi:10.1016/J.REDOX.2019.101127

101. Dean JM, Lodhi IJ. Structural and functional roles of ether lipids. Protein Cell. 2018;9: 196. doi:10.1007/S13238-017-0423-5

102. Tufarelli V, Colonna MA, Losacco C, Puvača N. Biological Health Markers Associated with Oxidative

Stress in Dairy Cows during Lactation Period. Metabolites. 2023;13. doi:10.3390/METABO13030405
103. Safa S, Kargar S, Moghaddam GA, Ciliberti MG, Caroprese M. Heat stress abatement during the postpartum period: effects on whole lactation milk yield, indicators of metabolic status, inflammatory cytokines, and biomarkers of the oxidative stress. J Anim Sci. 2019;97: 122–132. doi:10.1093/JAS/SKY408
104. Rödel HG, Jardim V, Rangassamy M, Jaravel L, Jacquet D, Monclús R, et al. Early life parameters and personality affect oxidative status during adulthood in an altricial rodent. Physiol Rep. 2022;10: 15427. doi:10.14814/PHY2.15427

Supporting information

S1 file. MRMs profiled in the screening phase

S2 file. Supplementary tables

S3 file. Description of significant lipids changes in each matrix under different light treatments within HF and CON diets

S4 file. Lipids modified by light treatment within the CON diet group

Fig1

Click here to access/download;Figure;Fig1.tif

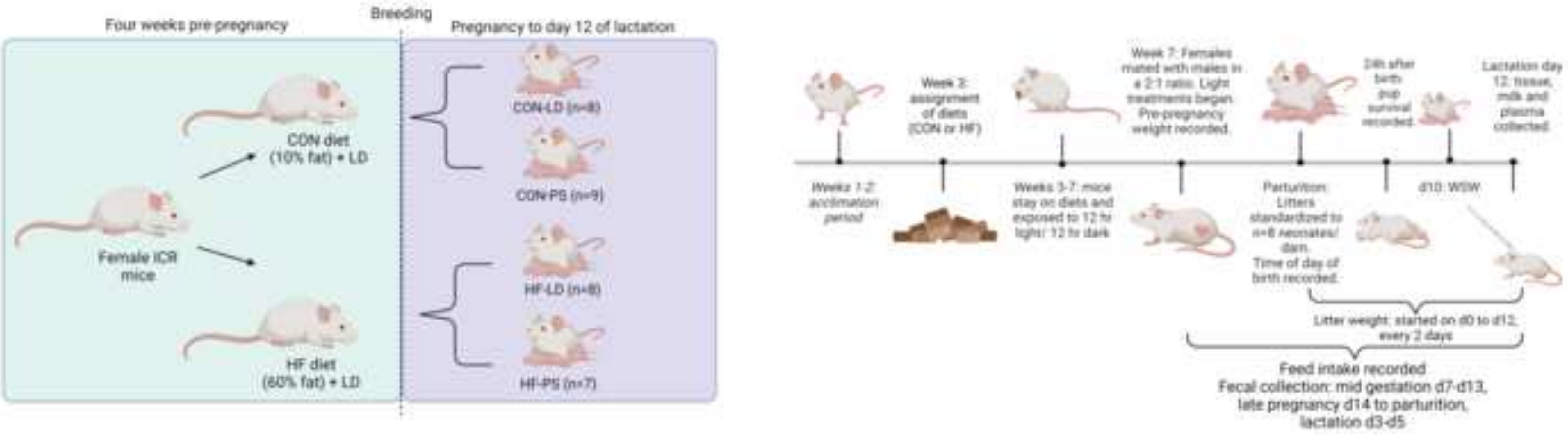

Fig2

Click here to access/download;Figure;Fig2.tif

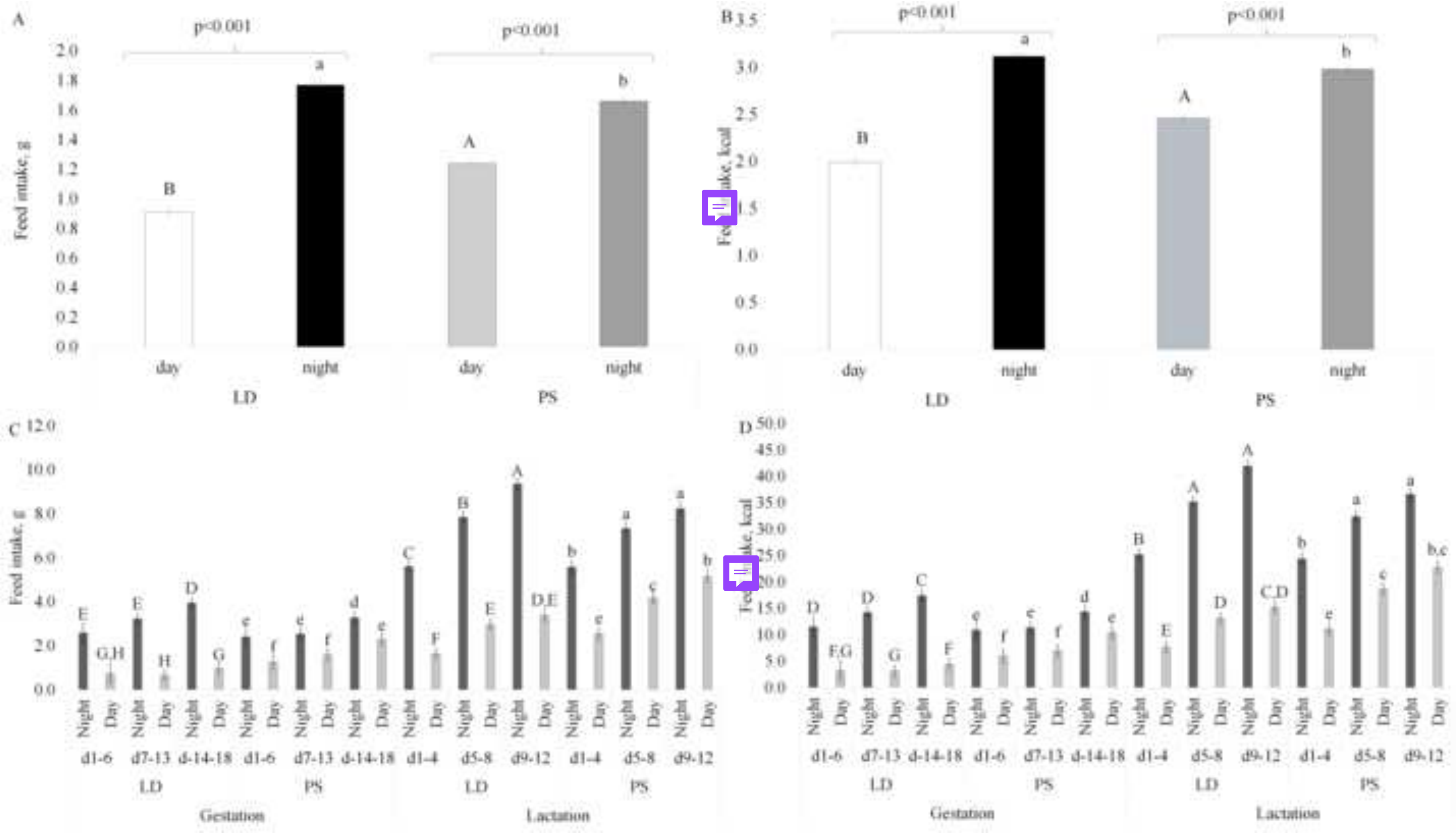

Fig3 Click here to access/download;Figure;Fig3.png

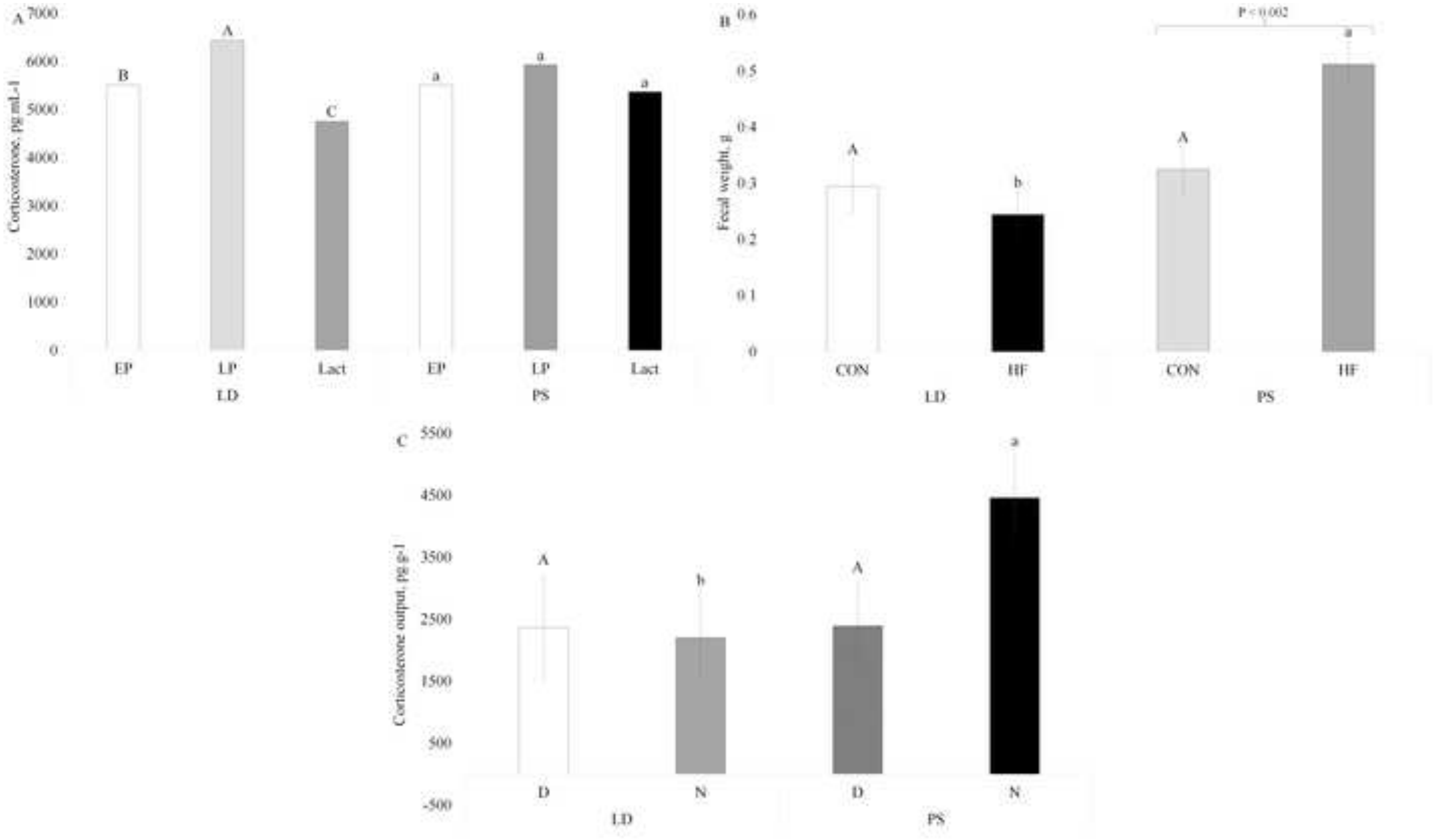

Fig4

Click here to access/download;Figure;Fig4.png

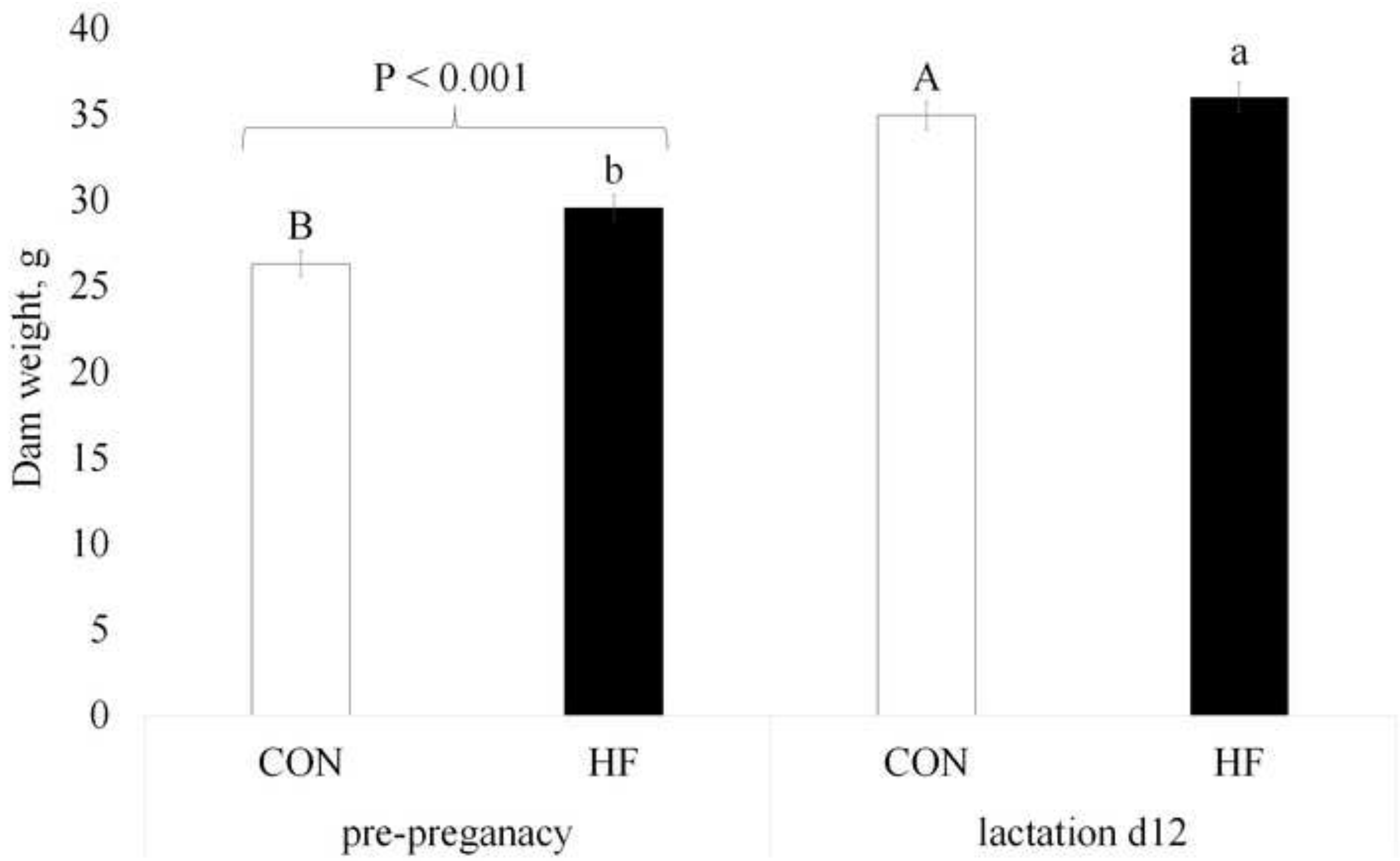

Fig5

Click here to access/download;Figure;Fig5.png

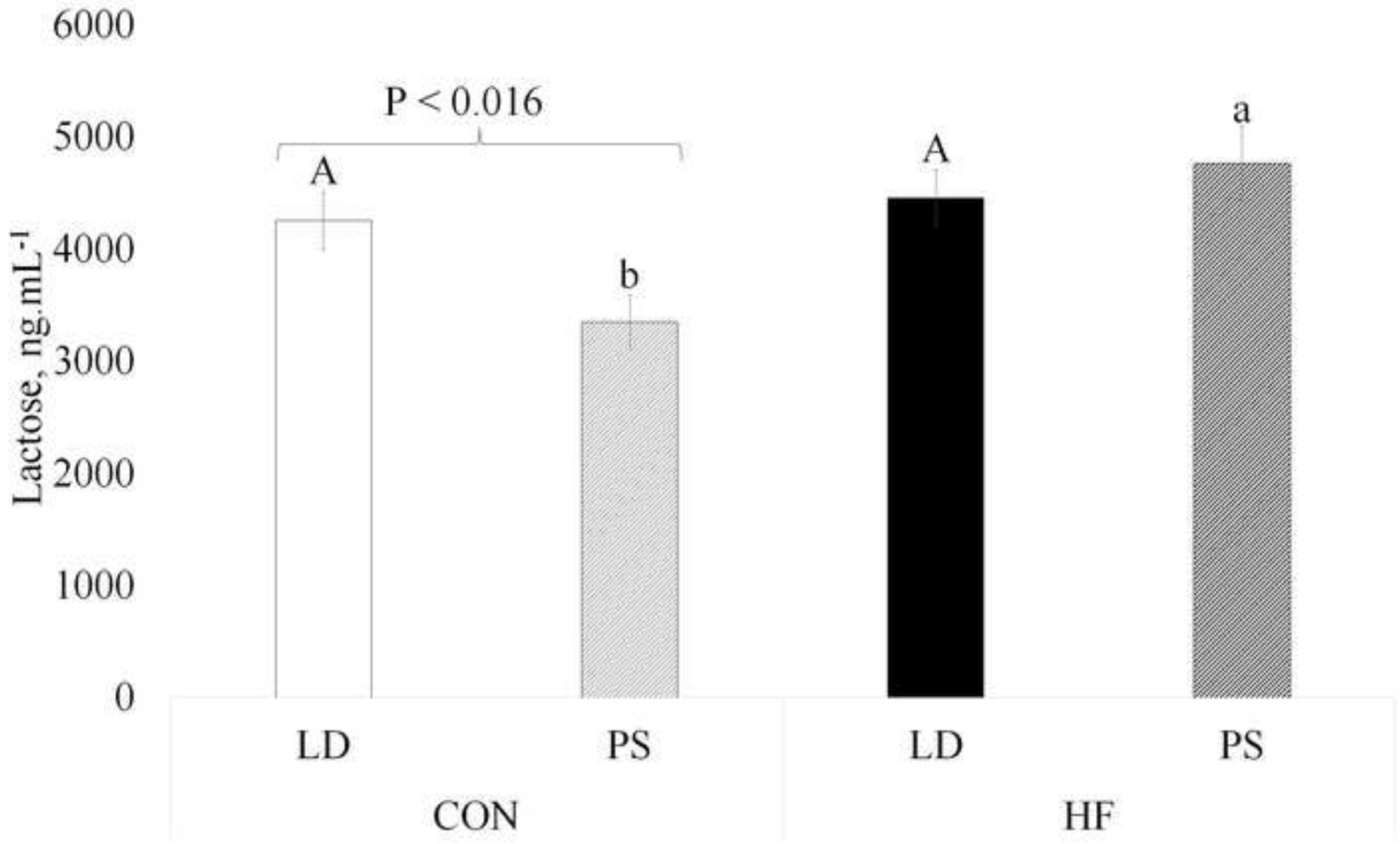

Fig6

Click here to access/download;Figure;Fig6.png

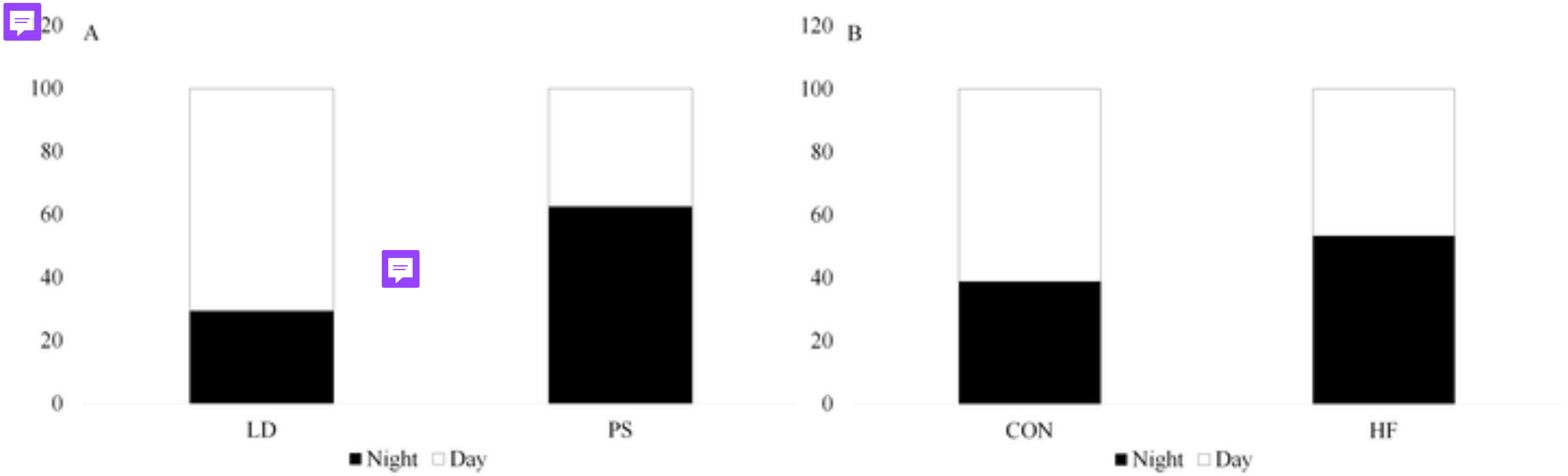

Fig7

Click here to access/download;Figure;Fig7.png

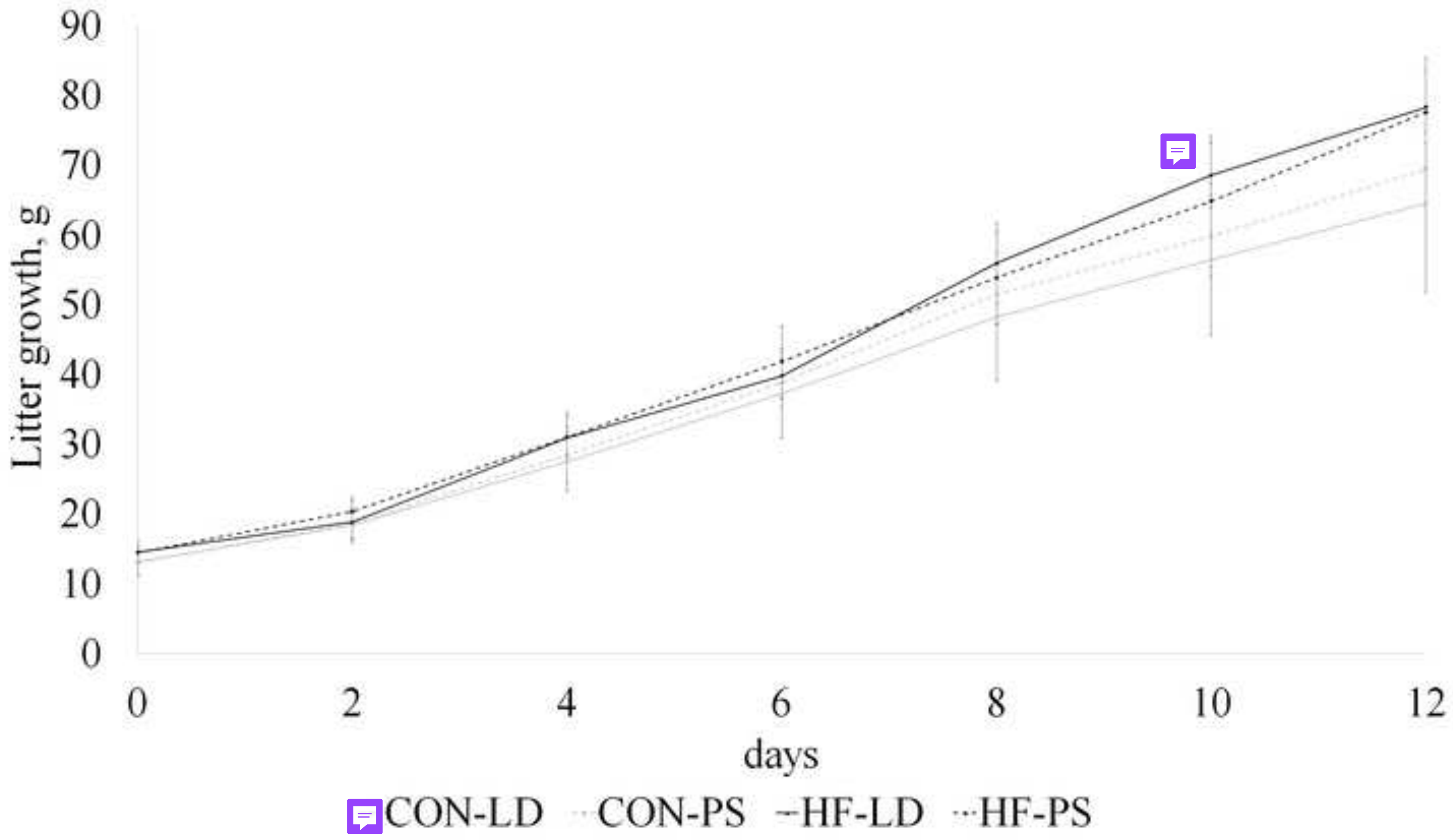

Fig8

Click here to access/download;Figure;Fig8.png

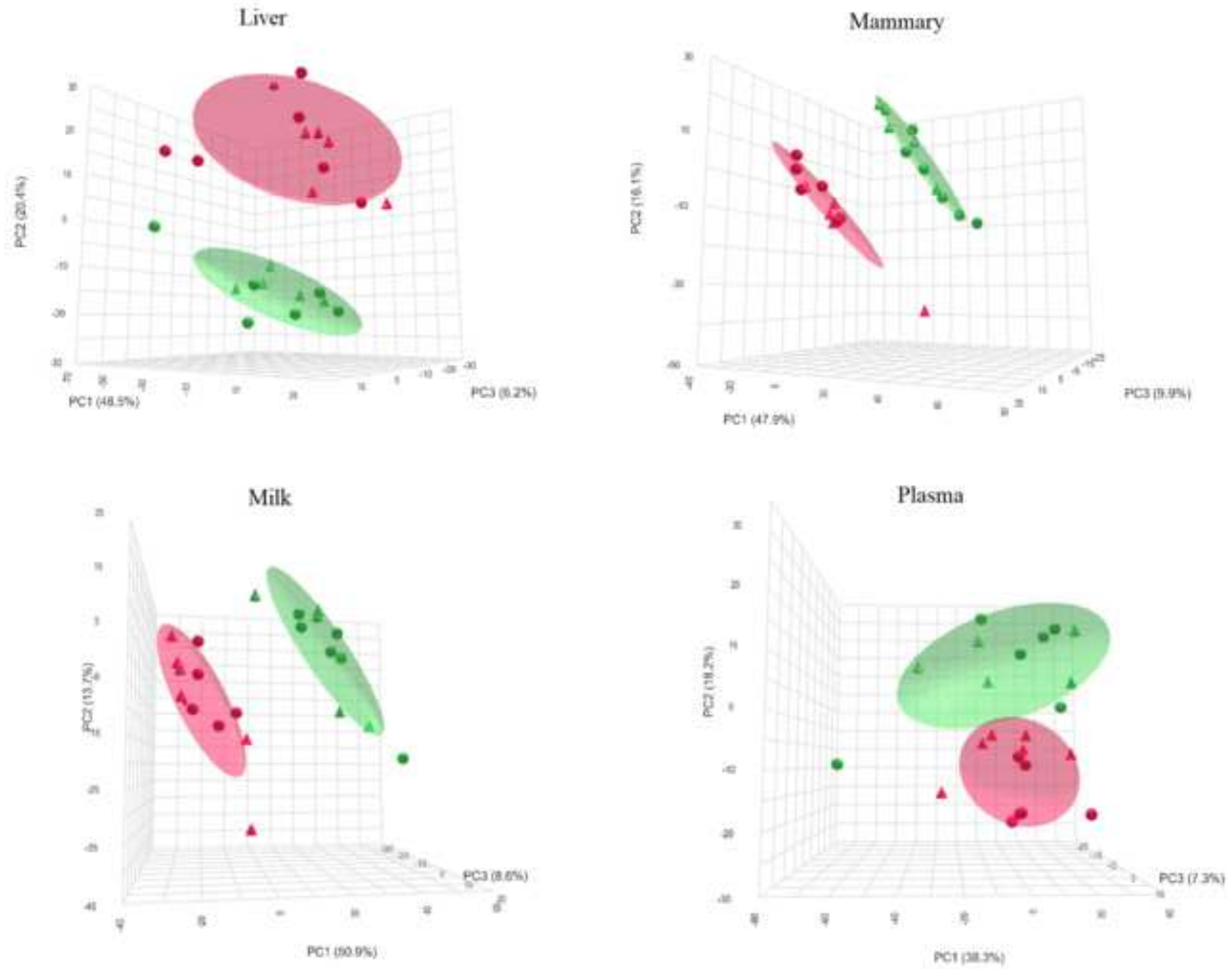

Fig9

Click here to access/download;Figure;Fig9.png

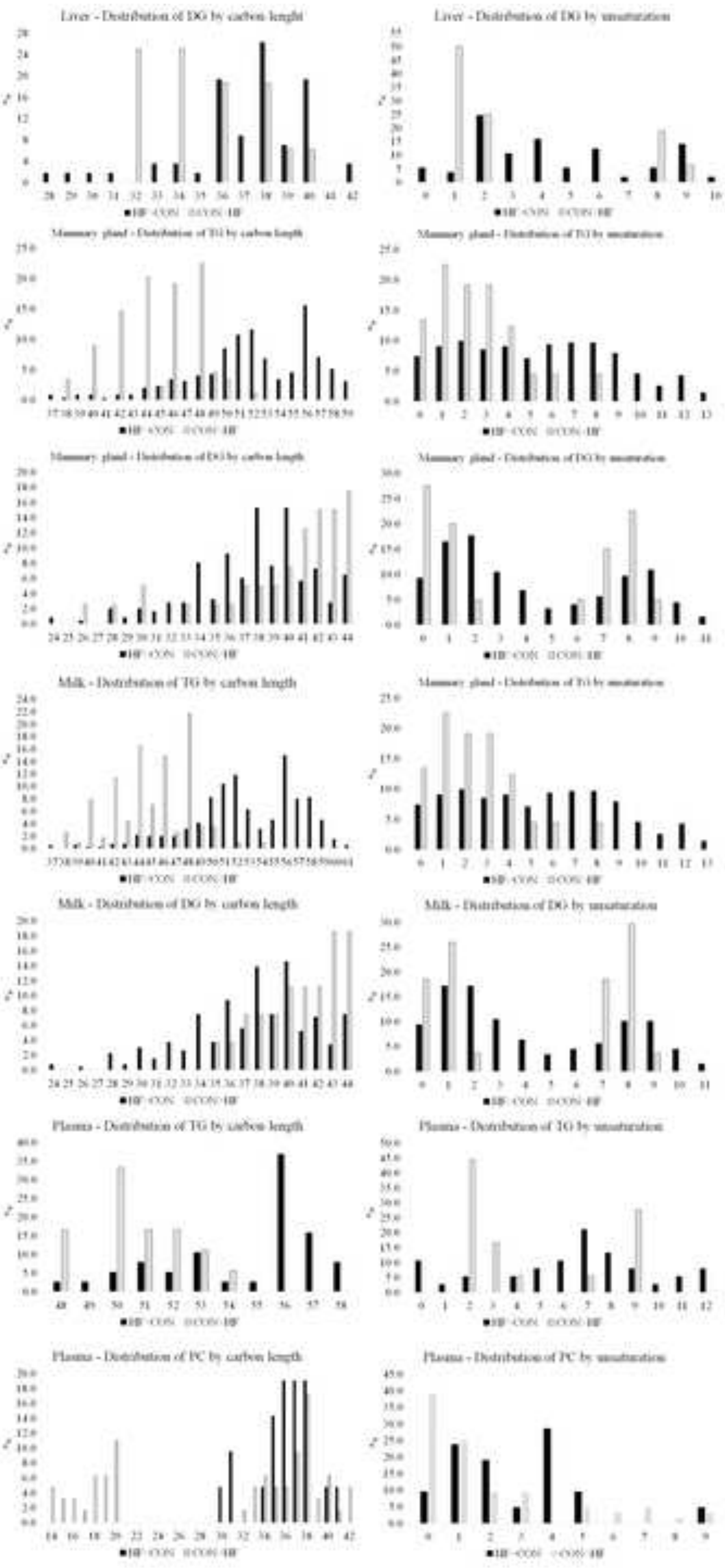

Fig10

Click here to access/download;Figure;Fig10.png

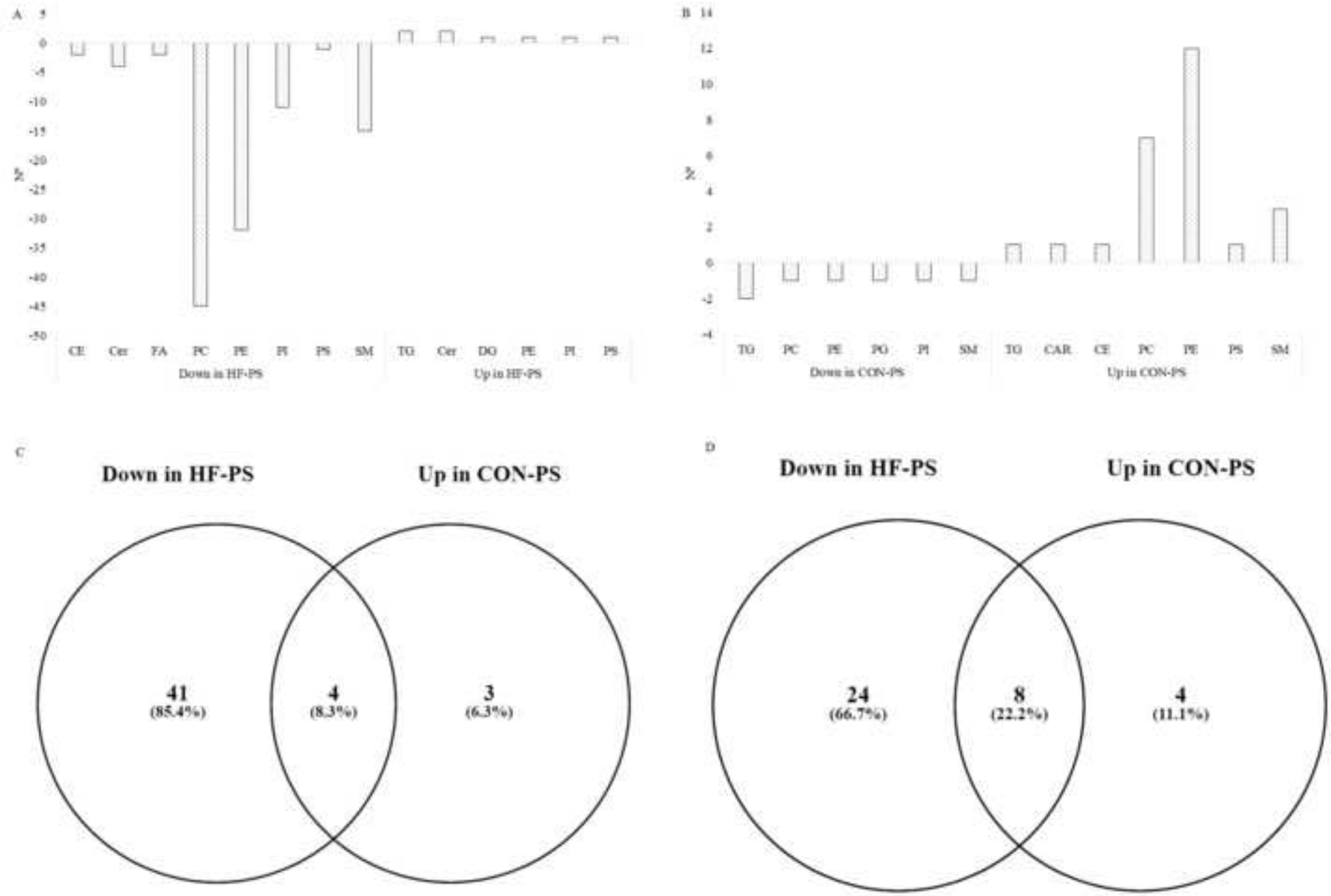

Click here to access/download
Supporting Information
S1_file.xlsx

S2_file

Click here to access/download
Supporting Information
S2_file.docx

S3_file

Click here to access/download
Supporting Information
S3_file.xlsx

S4_file

Click here to access/download
Supporting Information
S4_file.docx
